# Peripheral CD4^+^ naïve T cell remodeling and MMP1-associated inflammatory signatures in acute gouty arthritis

**DOI:** 10.3389/fimmu.2026.1828679

**Published:** 2026-05-26

**Authors:** Peng Zhang, Fangjie Zhu, Yunting Xiao, Zhengdong Shen, Chunxiao Fan, Peifeng Ke, Yao Xu, Chenguang Zhan, Xin Li, Xiaodong Wu, Liyan Mei, Kaixin Gao, Haifang Du, Xiumin Chen, Yang Jiao, Runyue Huang, Maojie Wang

**Affiliations:** 1 State Key Laboratory of Traditional Chinese Medicine Syndrome, The Second Affiliated Hospital of Guangzhou University of Chinese Medicine (Guangdong Provincial Hospital of Chinese Medicine), The Second Clinical Medical College of Guangzhou University of Chinese Medicine, Guangzhou, China; 2 State Key Laboratory of Dampness Syndrome of Chinese Medicine, The Second Affiliated Hospital of Guangzhou University of Chinese Medicine (Guangdong Provincial Hospital of Chinese Medicine), Guangzhou, China; 3 Guangdong Provincial Key Laboratory of Clinical Research on Traditional Chinese Medicine Syndrome, Guangzhou, China; 4 Department of General Practice (General Internal Medicine), Peking Union Medical College Hospital, Chinese Academy of Medical Sciences & Peking Union Medical College, Beijing, China

**Keywords:** CD4+ naïve T cells, gouty arthritis, granulocyte-like myeloid cells, immunocyte phenotype, matrix metallopeptidase 1

## Abstract

**Background:**

Gouty arthritis (GA) has long been considered a disease primarily driven by innate immune activation, whereas the contribution of adaptive immune remodeling remains incompletely understood.

**Methods:**

In this study, we integrated bidirectional Mendelian randomization (MR), CyTOF immune profiling, single-cell transcriptomic analysis, plasma proteomics, and prospective clinical follow-up analyses to investigate immune-associated features and recurrence-related inflammatory signatures in acute GA. Bidirectional two-sample MR analyses were performed using publicly available European ancestry GWAS datasets to evaluate genetic association patterns between 731 immune cell phenotypes and gout. Peripheral immune remodeling was subsequently characterized in Chinese cohorts using CyTOF analysis of PBMC samples from GA-A (n = 25), GA-R (n = 22), and healthy controls (n = 9), together with re-analysis of a public PBMC-derived single-cell RNA-seq dataset. Cell-cell communication, pathway enrichment, and differential expression analyses were performed to explore inflammatory programs associated with acute flares. Plasma inflammatory proteins were quantified using the Olink platform in independent training (n = 40) and validation (n = 64) cohorts, followed by exploratory recurrence risk stratification analyses incorporating independent validation, calibration analysis, and decision curve analysis.

**Results:**

Mendelian randomization analysis suggested associations between several immune traits and gout risk, including CD127⁺ CD4⁺ T cells and CD4⁺/CD8⁺ double-negative T cells. Mass cytometry of peripheral blood revealed expansion and functional remodeling of CD4⁺ naïve T cells during acute flares. Re-analysis of public single-cell RNA-seq data further showed that scRNA-seq-defined CD4⁺ naïve T cells exhibited inflammatory transcriptional features, including upregulation of S100A8/S100A9, and predicted interactions with granulocyte-like myeloid cells through MIF–CXCR2 and ANXA1–FPR1 signaling. Granulocyte-like myeloid cells displayed enhanced inflammatory and chemotactic signatures during acute GA. Plasma proteomic analysis identified elevated MMP1 and S100A12 levels during acute flares, both of which were associated with systemic inflammatory markers. Exploratory mediation analyses suggested that MMP1 may serve as a statistical mediator linking serum uric acid, systemic inflammation, and peripheral CD4⁺ naïve T cell abundance. In exploratory recurrence risk analyses, MMP1 showed exploratory potential for 12-week recurrence risk stratification, particularly within the GA-A subgroup (AUC = 0.8533).

**Conclusion:**

Together, these findings suggest that peripheral CD4⁺ naïve T cell remodeling and MMP1-associated inflammatory signatures may represent relevant features of acute GA and recurrence risk, while their mechanistic roles require further functional validation.

## Introduction

Gouty arthritis (GA) is a chronic inflammatory condition triggered by the deposition of monosodium urate (MSU) crystals in joint and periarticular tissues, resulting from sustained hyperuricemia (1). The disease manifests as acute flares with excruciating pain and swelling, and in advanced cases, progresses to chronic tophaceous gout, accompanied by persistent inflammation, joint destruction, and substantial morbidity (2, 3). Globally, the prevalence of gout ranges from 0.68% to 3.90% in population-based studies, with incidence rates peaking at 2.9 per 1, 000 person-years in certain populations (4–7).

Although therapies such as urate-lowering treatments (ULTs) and anti-inflammatory agents are effective in managing hyperuricemia and acute inflammation, many unresolved clinical challenges remain (8). These include the inability to predict acute flare, the mechanisms underlying disease progression from acute flares to chronic inflammation, and the failure to resolve persistent joint damage and tophus formation. Addressing these issues requires a deeper understanding of the underlying mechanisms. Most research on the pathogenesis of gout has concentrated on the innate immune response and urate metabolism, particularly the activation of the NLR family pyrin domain containing 3 (NLRP3) inflammasome by MSU crystals, which drives IL-1β release and neutrophil recruitment during acute flares (9). While critical, this mechanism alone does not fully account for the previously mentioned unresolved clinical challenges.

Recent research has started to delineate the contributions of adaptive immunity to GA. For instance, a single-cell sequencing study revealed that synovial fluid from acute gout was enriched in Th1 and Th17 cells, effector memory CD8 T cells, mucosal-associated invariant T cells, and macrophages as compared to healthy individuals (10). In addition, MSU crystals stimulates the production of Th17 cells and Th17-related inflammatory cytokines such as IL-17 *in vivo* (11), while anti-IL-17 antibody can attenuate joint symptoms, swelling and leukocytes infiltration to the inflamed tissue in GA animal model (11). Although current evidence suggests the involvement of T cell-mediated adaptive immune responses in GA, the identification of disease-associated T cell subsets, their functional states, and their potential relationships with inflammatory programs remain key unanswered questions.

To address these knowledge gaps, we combined genetic association analysis, peripheral immune phenotyping, public single-cell transcriptomic re-analysis, plasma proteomic profiling, and recurrence follow-up to explore adaptive immune remodeling and inflammatory signatures associated with GA. Bidirectional Mendelian randomization (MR) analysis suggested associations between several adaptive immunity-related immunophenotypes and gout susceptibility. Mass cytometry profiling of peripheral blood further revealed disease phase-associated immune remodeling, particularly involving CD4^+^ naïve T cells (TC01 subset). Re-analysis of public single-cell transcriptomic data showed that scRNA-seq-defined CD4^+^ naïve T cells displayed inflammatory transcriptional features and predicted interactions with granulocyte-like myeloid cells. Proteomic profiling revealed significantly elevated levels of matrix metallopeptidase 1 (MMP1) and S100 calcium binding protein A12 (S100A12) during the acute phase, both of which positively correlated with high-sensitivity C-reactive protein (hCRP) and erythrocyte sedimentation rate (ESR). In addition, MMP1 showed exploratory potential for short-term recurrence risk stratification. Together, these analyses provide exploratory evidence linking peripheral adaptive immune remodeling and MMP1-associated inflammatory signatures with acute GA and recurrence risk.

## Materials and methods

### Bidirectional two-sample Mendelian randomization analysis

**Figure 1:**
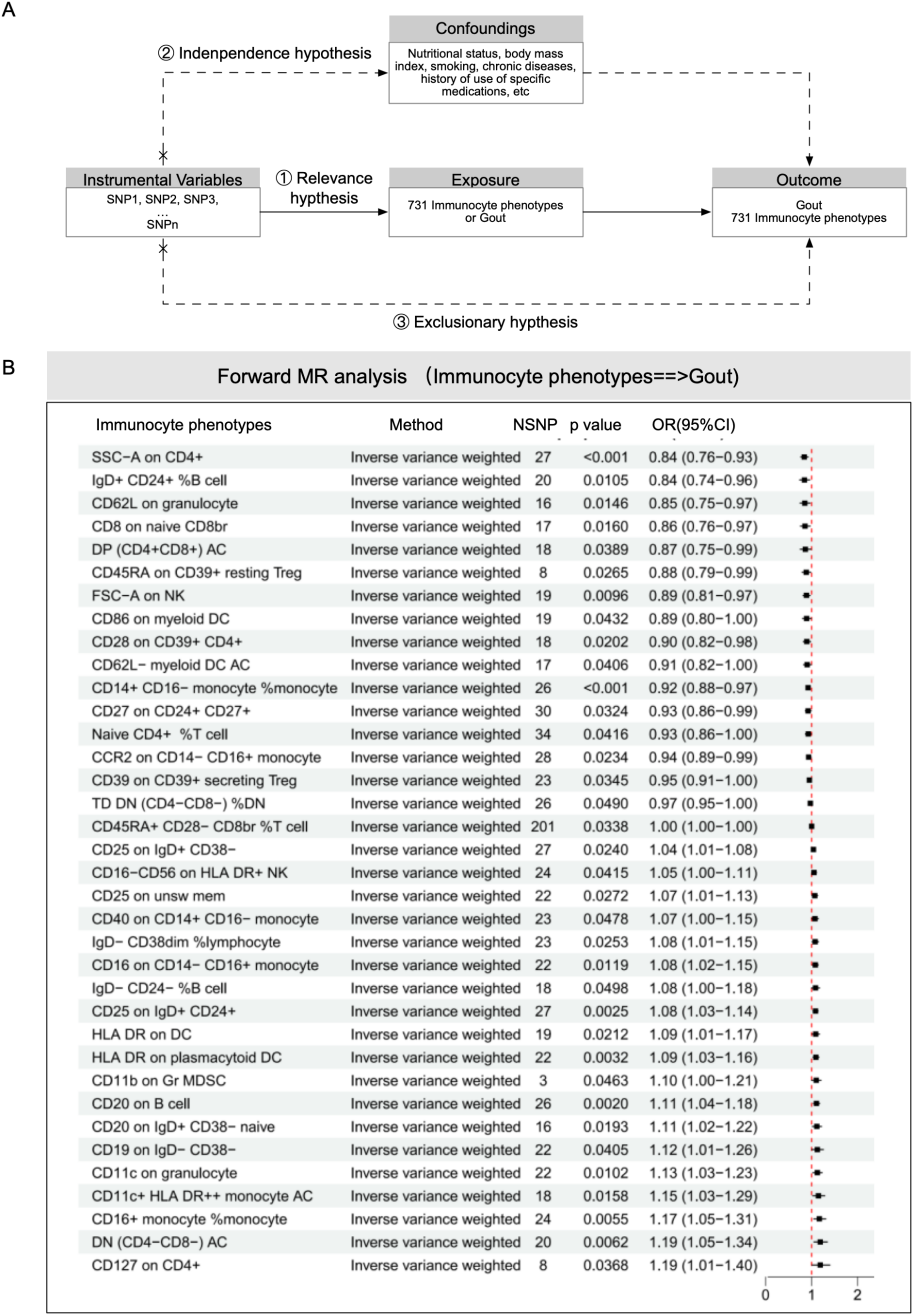
Mendelian randomization analysis design and forward analysis result. **(A)** The MR analysis framework. (1) Relevance hypothesis: IVs were strongly associated with exposure factors; (2) Independence hypothesis: IVs should not be influenced by known or unknown confounders; and (3) Exclusionary hypothesis: IVs influenced outcome factors only through exposure factors. **(B)** Forward MR analysis assessing genetic associations of 731 immune cell phenotypes with gout using the inverse-variance weighted (IVW) method.

We assessed the genetic associations between 731 immunocyte phenotypes and gout using bidirectional two-sample MR analysis. Our MR analysis was conducted in three steps according to the following framework: (1) Relevance hypothesis: Instrumental Variables (IVs) were strongly associated with exposure factors; (2) Independence hypothesis: IVs should not be influenced by known or unknown confounders; and (3) Exclusionary hypothesis: IVs influenced outcome factors only through exposure factors (12, 13) (**Figure 1A**). Data on 731 immunocyte phenotypes were obtained from the GWAS Catalog (accession numbers GCST90001391 to GCST90002121, https://www.ebi.ac.uk/gwas/downloads/summary-statistics), based on a cohort of 3, 757 individuals of European ancestry. GWAS summary statistics for 1, 699 gout cases and 216, 239 controls were obtained from the Finnish database (https://storage.googleapis.com/finngen-public-data-r5/summary_stats/finngen_R5_GOUT_STRICT.gz).

Single nucleotide polymorphisms (SNPs) were selected based on a significance threshold of p < 1×10^-5^. To reduce linkage disequilibrium effects, an r² threshold of 0.001 and a genomic distance of 10, 000 Kb were applied. SNPs associated with confounders were excluded via the PhenoScanner V2 database. The specific confounders we targeted included nutritional status, body mass index (BMI), smoking behavior, chronic diseases (e.g., chronic kidney disease and hypertension), and history of use of specific medications (e.g., diuretics or urate-lowering therapies). SNPs significantly associated with these traits at a genome-wide level were excluded to minimize potential bias arising from pleiotropic pathways. Instrument strength was confirmed by calculating the F-statistic (F = β^2^/SE^2^); SNPs with F < 10 were excluded. SNPs consistent with the study hypotheses were retained, and the exposed and resultant datasets were merged. To ensure data integrity, palindromic sequences were removed, resulting in the final set of IVs for the exposure.

MR analyses were conducted in R 4.3.1 using the TwoSampleMR package (version 0.6.8) and included five methods: inverse variance weighted (IVW), weighted median, simple mode, weighted mode, and MR-Egger regression. IVW, serving as the primary method, was weighted by the inverse of SE² without an intercept term. Sensitivity analyses were performed to evaluate the robustness of the Mendelian randomization results. Heterogeneity among instrumental variables was assessed using Cochran’s Q statistics derived from both the inverse variance weighted (IVW) and MR-Egger models, with p < 0.05 indicating significant heterogeneity. Horizontal pleiotropy was evaluated using the MR-Egger intercept test, and leave-one-out analyses were conducted to assess the influence of individual SNPs on the overall causal estimates. Results were reported as odds ratios (ORs) with 95% confidence intervals (CIs), with significance set at p < 0.05.

### Human sample collection

According to the protocol approved by the Ethics Committee of Guangdong Provincial Hospital of Chinese Medicine (B2020-193-01), peripheral blood mononuclear cells (PBMCs) and plasma were collected from patients with GA meeting the 2015 Gout Classification Criteria, an American College of Rheumatology/European League Against Rheumatism collaborative initiative (14). Healthy individuals were enrolled according to the ethics protocol with ID of BF2021-235-01. All laboratory test results were sourced from the hospital’s outpatient electronic system. Blood samples were obtained using K2 EDTA tubes (BD Vacutainer) and processed by density-gradient centrifugation with Lymphocyte-H Cell Separation Media (Cedarlane). The isolated PBMCs were preserved in a cryopreservation solution containing 90% fetal bovine serum (Gibco) and 10% dimethyl sulfoxide (DMSO) (Sigma) at a density of 0.5–1 million cells/mL and stored in liquid nitrogen. Written informed consent was obtained from all participants included in training and validation cohorts.

### Antibodies staining, acquisition, and bio-informatics analysis of mass cytometry data

Details of the antibodies used in this study, including names, clone numbers, catalog numbers, brands, labeled metal isotopes, and concentrations, are listed in **Supplementary Table 1**. Antibody labeling procedures were performed as described in our previous publication (15). Data acquisition was conducted using the Helio3 CyTOF mass spectrometer (Fluidigm).

Raw CyTOF data were first normalized and debarcoded prior to downstream analysis. Manual gating was subsequently performed in FlowJo software (version 10.8.1) following a sequential gating strategy. Briefly, cellular events were initially identified based on high Ir191 DNA and Ir193 DNA signals to exclude debris and non-cellular impurities. Viable cells were then selected by excluding cisplatin-positive events using the Pt194Di (194 cisplatin) channel. Doublets and cell aggregates were further removed based on Event Length gating to retain singlet cells. Residual normalization beads were excluded using the Ce140Di channel. After obtaining viable singlet immune cells, CD45^+^CD66b^-^ cells were gated for downstream immune cell population analysis. The complete CyTOF gating strategy is provided in **Supplementary Figure 2**.

We utilized an advanced bioinformatics pipeline based on the CyTOF workflow (version 3) as outlined in F1000Research (16). R packages such as flowWorkspace, flowCore, and CATALYST facilitated data manipulation and analysis. A random subset of 20, 000 events per sample was selected for consistency, and the data were preprocessed into a SingleCellExperiment object using the prepData function. Clustering algorithms were applied to the SCE object to identify immune cell populations, with CD66b^-^CD45^+^ lymphocytes isolated into a separate SCE object for detailed profiling. Results were visualized using heatmaps, bar plots, box plots, and t-SNE plots, with marker expression overlaid to provide functional insights. Major immune cell populations were reclassified and consolidated based on marker expression, while specific compartments, such as T cells (CD45^+^CD3^+^), underwent additional clustering for further analysis.

### Single-cell RNA-seq data analysis

Raw gene expression matrices were obtained from the GEO database (GSE211783) and processed using the Seurat R package (v4.3.0). Cells from six peripheral blood samples, comprising gout acute flare (GA-A, n=3) and remission (GA-R, n=3) conditions, were loaded via Read10X and filtered based on gene count (200–6000 genes per cell) and mitochondrial content (<10%). SCTransform was applied to normalize and variance-stabilize data from each sample individually. For batch correction and integration, 3, 000 highly variable genes were selected, and integration anchors were identified using FindIntegrationAnchors. Integrated data were then dimensionally reduced via PCA, followed by clustering (FindNeighbors, FindClusters) and UMAP visualization (RunUMAP). Dimensionality selection was informed by elbow plot inspection.

Cell type annotation was initially performed using the SingleR package with the Human Primary Cell Atlas (HPCA) reference dataset. To improve the robustness of immune cell annotation, particularly for myeloid and granulocyte-associated populations in PBMC-derived data, additional reference datasets including MonacoImmuneData, BlueprintEncodeData, and DatabaseImmuneCellExpressionData (DICE) were further incorporated for comparative annotation analysis. Annotation results across references were compared at the cluster level and manually refined based on canonical marker gene expression patterns. Canonical lineage markers used for manual curation included CCR7, IL7R, LTB, and MAL for CD4^+^ naïve T cells; NKG7 and GNLY for NK cells; CD79A and MS4A1 for B cells; LYZ, FCGR3A, CD14, and LST1 for monocyte/myeloid populations; and FCGR3B, CSF3R, S100A8, S100A9, CXCR2, and CEACAM8 for granulocyte-associated myeloid populations. Because conventional high-density neutrophils are typically underrepresented in PBMC preparations, clusters showing partial enrichment of granulocyte-associated signatures were conservatively annotated as “granulocyte-like myeloid cells” rather than canonical neutrophils. Clusters with low annotation confidence, inconsistent lineage assignment across reference datasets, or sparse representation (<50 cells) were excluded from downstream analyses. The final annotation framework adopted in this study is summarized in **Supplementary Table 2**.

Subsequent analyses were conducted on the filtered annotated object. Marker genes for each cluster were identified using FindAllMarkers with Wilcoxon rank-sum testing on SCT-normalized data, requiring log2 fold-change > 0.25 and adjusted p-value < 0.01. Top marker genes were visualized via dot plots.

Comparative analyses focused on specific immune cell subsets, including CD4^+^ naïve T cells and granulocyte-like myeloid cells. Differential gene expression (DGE) between GA-A and GA-R was assessed within the subset using FindMarkers. Genes with |log2FC| > 0.585 and adjusted p < 0.001 were considered significant. Functional enrichment of up- and downregulated genes was performed using KEGG pathway analysis via the clusterProfiler and org.Hs.eg.db packages. For broader pathway context, gene set enrichment analysis (GSEA) was conducted using fgsea with MSigDB hallmark gene sets (via msigdbr).

To characterize intercellular communication, we used the CellChat package. A Seurat object was converted to a CellChat object, and the built-in human ligand-receptor interaction database was used. Communication probability was computed across identified cell types, and signaling networks were inferred using the computeCommunProb, aggregateNet, and netVisual_circle functions. Key signaling pathways showing altered activity between GA-A and GA-R were highlighted. All plots, including UMAP, dot plots, bar plots, volcano plots, KEGG/GSEA enrichment maps, and communication networks, were visualized using a combination of ggplot2, scplotter, EnhancedVolcano and enrichplot.

### O-link analysis and data processing

A total of 138 plasma samples from two cohorts were collected at enrollment, stored at -80 °C, and thawed on ice before analysis. After thorough mixing by pipetting, the samples were randomized and plated onto 96-well PCR microplates. Proteomic measurements were performed using the O-Link Proximity Extension Assay (PEA) technology, which employs qPCR for the simultaneous quantification of a pre-designed panel of proteins in each sample (17). Specifically, the O-Link^®^ Target 96 Inflammation Panel was used in this study, with proteomic assays, data normalization, and quality control conducted by BGI, China. Proteins with linear normalized protein expression (NPX) values below the detection limit in ≥20% of samples were excluded from further analysis, resulting in a final dataset of 75 proteins.

### Construction and validation of models for predicting gout recurrence

Multiple logistic regression models were developed to predict gout relapse within 12 weeks. Predictor variables included body mass index (BMI), laboratory test parameters, and NPX levels of selected immune molecules, 78 variables in total. The outcome variable was recurrence status, classifying patients into recurrence and non-recurrence groups based on whether a flare occurred within 12 weeks. The modeling dataset was derived from the training cohort (n = 40), and external validation was performed using the independent validation cohort (n = 64). Feature selection was performed exclusively within the training cohort using LASSO regression with ten-fold cross-validation to identify non-zero variables while reducing overfitting. Eight non-zero variables were retained after LASSO selection. Among these candidates, five variables were further prioritized based on their discriminatory performance (AUC) and significant between-group differences within the training cohort. Subsequently, logistic regression models were constructed using different combinations of these five variables in the training cohort and evaluated in the independent external validation cohort. Model performance was assessed using AUC and classification accuracy.

### Multi-model validation of MMP1 for predicting GA flare within 12 weeks

To evaluate the discriminatory performance of MMP1 for GA relapse within 12 weeks, a workflow set comprising five machine learning models, XGBoost, decision tree, logistic regression, K-Nearest Neighbors (KNN), and random forest, was implemented. The predictor variable was the NPX value of MMP1, and the outcome variable was the relapse status within 12 weeks, categorizing patients into recurrence and non-recurrence groups.

The modeling dataset was derived from the training cohort, while the validation dataset was obtained from the external validation cohort. Specifically, the dataset was imported, and a preprocessing recipe was defined with group (recurrence vs. non-recurrence) as the response variable and MMP1 as the predictor. All five models were configured using the tidymodels framework with appropriate engines and classification modes. A workflow set was constructed by combining the preprocessing recipe with each machine learning model. Resampling was conducted via bootstrapping with 1, 000 iterations to improve the stability of model performance estimation, and the resulting predictions were retained for subsequent analysis. For tree-based models and XGBoost, hyperparameters were tuned using the tidymodels framework with grid-based optimization. The evaluated hyperparameters included tree depth, minimum node size, learning rate, and number of trees where applicable. For KNN models, the number of neighbors was optimized. Logistic regression was included as a baseline interpretable model given the relatively small sample size and limited number of predictors. Model performance was evaluated using accuracy, Brier score, and AUC. Results were ranked and visualized accordingly.

To evaluate the potential confounding effect of disease phase, additional sensitivity analyses restricted to the GA-A subgroup were performed using the same modeling framework. Decision curve analysis (DCA) was conducted to evaluate the potential relevance of the model by quantifying the net benefit across a range of threshold probabilities. Net benefit was calculated according to the standard formula: Net benefit = (TP/N) − (FP/N) × (pt/(1-pt)), where TP represents true positives, FP represents false positives, N represents the total number of patients, and pt represents the threshold probability. Calibration analysis was performed to evaluate the agreement between predicted probabilities and observed outcomes in the validation cohort. Predicted probabilities were obtained from the logistic regression model and assessed using calibration curves generated with the caret package in R. The validation cohort was stratified into grouped probability intervals, and the mean predicted probability was compared with the observed event rate within each group. Calibration intercept and calibration slope were additionally calculated to quantitatively assess model calibration. To avoid infinite values during logit transformation, predicted probabilities were constrained within the range of 1×10^-6 to 1−1×10^-6. The calibration slope and intercept were estimated by fitting a logistic regression model using the observed outcome as the dependent variable and the logit-transformed predicted probability as the independent variable. An ideal calibration performance was defined as a calibration intercept close to 0 and a calibration slope close to 1.

### Mediation linkage inference

We first screened the exposure variable and mediator variables that were significantly associated with the specific outcome variable (ESR or hCRP) by mediation in R package mediation (version 4.5.0) (p value of total effect <0.05). Simple mediation analysis was performed using the mediate function to evaluate statistical indirect associations between mediator variables and outcome variables.

### MSU stimulation assay in fibroblast-like synoviocytes

Rheumatoid arthritis fibroblast-like synoviocytes (RA-FLS) were cultured in DMEM supplemented with 10% fetal bovine serum and 1% penicillin-streptomycin under standard conditions (37 °C, 5% CO_2_). Cells were stimulated with different concentrations of monosodium urate (MSU) crystals for 24 hours. Total RNA was extracted using TRIzol reagent, and cDNA synthesis was performed using reverse transcription kits according to the manufacturer’s instructions. MMP1 mRNA expression levels were quantified by quantitative real-time PCR and normalized to GAPDH expression using the 2^-ΔΔCt method. The primer sequences are AAAATTACACGCCAGATTTGCC (Forward) and GGTGTGACATTACTCCAGAGTTG (Reverse).

### Correlation analysis

The correlation coefficients and their associated p values were calculated using the Spearman method and scatter plots were generated using ggplot2 package. Points were colored and shaped according to cohorts, with custom-defined colors. Linear trend lines with 95% confidence intervals were added for each condition.

### Statistics

Statistical analyses were done by using GraphPad Prism v10 or R statistical package as indicated. Specific statistical methods used for each dataset are detailed in the legends of the corresponding Figures and Tables. P < 0.05 were considered statistically significant.

### Data availability statement

The O-Link data was normalized and the NPX data is provided in **Supplementary Table 3**. CyTOF data can be requested from the corresponding authors upon reasonable request and with appropriate permissions.

## Results

### Adaptive immune cell phenotypes are genetically associated with gout risk in MR analysis

Although gout has long been attributed to innate immune activation, emerging studies implicate adaptive immunity in disease pathogenesis. To evaluate genetic evidence linking specific immune phenotypes with gout risk, particularly those of adaptive immune origin, we performed a bidirectional two-sample MR analysis using publicly available datasets from the GWAS Catalog and FinnGen database, following the analytical framework illustrated in **Figure 1A**.

Using the IVW method as the primary analytical approach, we identified 35 immune cell phenotypes genetically associated with gout risk (**Figure 1B**). Of these, 19 immunophenotypes were associated with higher odds ratio (OR) of gout (OR > 1), while 16 displayed lower odds of gout (OR < 1). As expected, several innate immune phenotypes, including CD11c^+^ HLA-DR^++^ monocytes, CD16^+^ monocytes, and CD40^+^ classical monocytes, showed positive associations with disease. However, notably, multiple adaptive immune phenotypes also demonstrated significant positive associations, including: Double-negative T (DNT) cell absolute count (OR = 1.19, p = 0.0062), CD20 expression on B cells (OR = 1.11, p = 0.0020), CD127 expression on CD4^+^ T cells (OR = 1.19, p = 0.0368) and HLA-DR expression on dendritic cells (DCs and pDCs) (OR = 1.09, p < 0.05).

To further explore bidirectional MR association patterns, we performed reverse MR analysis using gout as the exposure. Only CD45RA expression on CD39^+^ resting regulatory T cells (Tregs) showed a significant association (OR = 0.92, p = 0.0033), consistent with its association with lower odds of gout observed in the forward analysis (OR = 0.88, p = 0.0265). These findings suggest a potential bidirectional genetic association pattern between gout and immunoregulatory phenotypes. Sensitivity analyses, including Cochran’s Q test and MR-Egger intercept testing, revealed no evidence of heterogeneity or horizontal pleiotropy (**Supplementary Tables 4**, **5**), supporting the validity of the identified associations. Collectively, these findings suggest potential genetic links between adaptive immune traits and gout susceptibility, warranting further exploratory immune profiling.

### Lymphocyte composition and functional states are dynamically altered during different phases of gout arthritis

To better understand the immune cell remodeling in GA, we profiled PBMCs from patients during the acute (GA-A, n = 25) and remission (GA-R, n = 22) phases using 41-marker mass cytometry, alongside 9 matched healthy controls (HC) (clinical features in **Supplementary Table 6**). Key immune populations, including B cells, CD4^+^ and CD8^+^ T cells, DNT and double-positive T (DPT) cells, γδ T cells, NKT cells, NK cells, and myeloid cells, were identified by canonical markers (**Supplementary Figure 2A**). DNT cell proportions were significantly increased in both GA-A and GA-R relative to HC, while CD4^+^ T cell frequencies were elevated during the acute phase compared to remission (**Supplementary Figure 2B**).

To assess functional differences, we analyzed the expression of migration and activation markers. GA-A-associated lymphocytes, including CD4^+^ and CD8^+^ T cells, NKT cells, and B cells, exhibited increased CCR7, suggesting enhanced lymphoid tissue homing during active inflammation (**Supplementary Figure 2C**). In GA-A, DNT cells exhibited upregulated CCR2 expression but downregulated levels of pCREB, a transcription factor associated with immune quiescence. Similarly, myeloid cells also showed elevated CCR2 expression, suggesting enhanced migratory potential driven by chemokines such as CCL2, CCL7, CCL8, and CCL13. B cells in GA-A also displayed elevated CXCR5 and HLA-DR, indicative of increased responsiveness to CXCL13 and enhanced antigen-presenting capacity (**Supplementary Figure 2C**). These findings point to disease phase-specific immune remodeling characterized by altered trafficking and functional activation.

### T cell subsets undergo compositional and functional reshaping in gout arthritis

**Figure 2:**
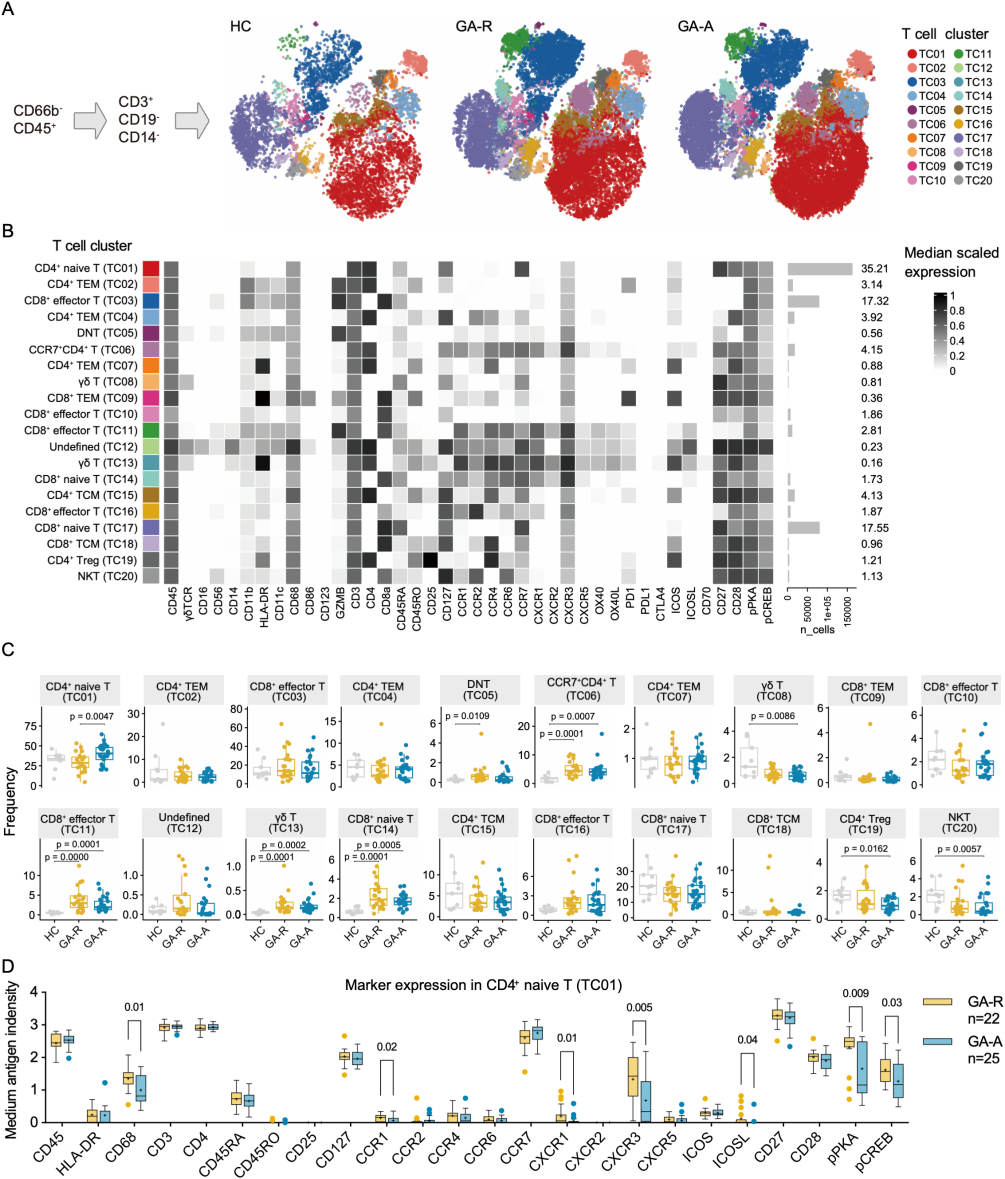
T cell landscape in GA and marker expression profile of CD4^+^ naïve T cells. **(A)** tSNE plots displaying the distribution of CD45^+^CD3^+^CD19^-^CD14^-^ T cell subsets across 20 distinct T cell clusters (TC01-TC20) in HC, GA-R, and GA-A. **(B)** The heatmap displays the median expression levels of markers across 20 identified T cell subsets. The bar plot on the right shows the average proportion of each subset across all samples. **(C)** Boxplots demonstrating the relative frequencies of T cell subsets across HC (n=9), GA-R (n=22) and GA-A (n=25). Statistical significance was evaluated using the Kruskal-Wallis test, with corresponding p-values indicated for each comparison. **(D)** The bar chart depicting the expression levels of representative markers in CD4^+^ naïve T cells (TC01) subsets between GA-R and GA-A groups. Statistical significance was evaluated using the multiple Mann-Whitney tests, with corresponding p-values indicated for each comparison. GA-A, gout patients at acute stage; GA-R, gout patients at remission stage; HC, healthy individuals; TC, T cell cluster; TCM, central memory T cells; TEM, effector memory T cells; NKT, natural killer T cells.

T cells play central roles in immune regulation, yet their subset composition and functional states in GA remain poorly characterized. To fill this knowledge gap, we further gated CD45^+^CD3^+^CD19^-^CD14^-^ T cells to comprehensively profile peripheral T cells and delineate their phenotypic and functional remodeling across different disease stages. T-cell subsets identified by CyTOF were annotated based on canonical surface marker expression. We identified 20 distinct T cell subsets (TC01-TC20) through t-SNE clustering (**Figure 2A**). These included CD4^+^ and CD8^+^ T cells, γδ T cells, DNT cells, and NKT cells, with marker-based classification and proportional representation shown in **Figure 2B**. CD4^+^ naïve T cells were defined as CD3^+^CD4^+^ T cells with high CCR7 and CD45RA expression and low expression of effector/memory-associated markers. Comparative analysis revealed that CCR7^+^CD4^+^ T cells (TC06), CD8^+^ effector T cells (TC11), γδ T cells (TC13), and CD8^+^ naïve T cells (TC14) were elevated in GA-A and GA-R compared to HC (**Figure 2C**). In contrast, γδ T cells (TC08), CD4^+^ regulatory T cells (TC19), and NKT cells (TC20) were significantly reduced, particularly during the acute phase. Of note, CD4^+^ naïve T cells (TC01), the most abundant subset, were increased in GA-A compared to GA-R. Functional profiling of TC01 revealed decreased expression of CD68, CXCR1, CXCR3, pPKA, and pCREB in GA-A, suggesting impaired migratory and transcriptional activity during acute flares (**Figure 2D**). Together, these results demonstrate that GA is associated with a broad reshaping of T cell subsets, marked by both quantitative shifts and altered functional states.

### CD4^+^ naïve T cells exhibit pro-inflammatory programming and granulocyte-like myeloid cells interactions during acute flares

**Figure 3:**
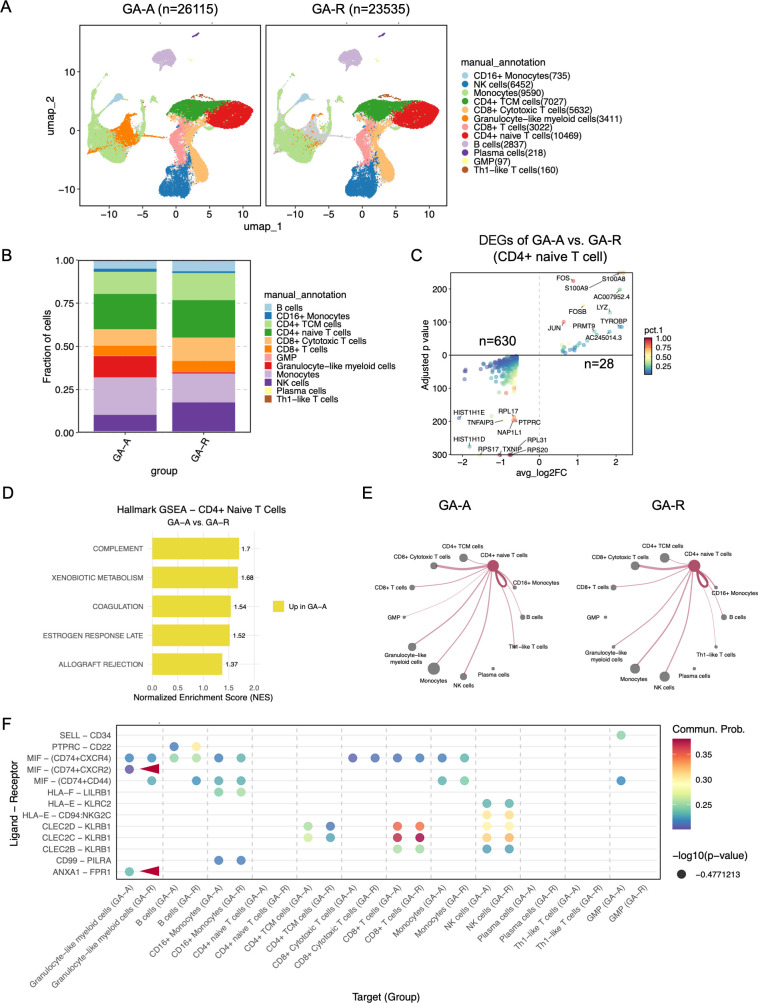
Single-cell transcriptomic analysis reveals immune remodeling and altered intercellular communication patterns in GA-A and GA-R. **(A)** UMAP plot showing the immune landscape of PBMCs from three GA patients during acute (GA-A) and remission (GA-R) phases. Data were obtained from the GEO dataset (GSE211783). **(B)** Stacked bar plot showing the relative proportions of annotated immune cell subsets in GA-A and GA-R samples. **(C)** Volcano plot illustrating DEGs in CD4^+^ naïve T cells between GA-A and GA-R groups. **(D)** Hallmark gene set enrichment analysis (GSEA) of CD4^+^ naïve T cells comparing GA-A versus GA-R. Bars indicate normalized enrichment scores (NES) for significantly enriched pathways in GA-A. **(E)** Cell-cell communication networks showing the interaction strength between CD4^+^ naïve T cells and other immune cell subsets in GA-A and GA-R, respectively. **(F)** Dot plot showing selected ligand-receptor interaction pairs involving CD4^+^ naïve T cells and other immune cell populations. Predicted MIF–CXCR2 and ANXA1–FPR1 signaling interactions were enriched in granulocyte-like myeloid cells in GA-A. NK, natural killer cells; DEGs, differentially expressed genes; GSEA, gene set enrichment analysis; NES, normalized enrichment scores; CXCR, C-X-C motif chemokine receptor.

CD4^+^ naïve T cells are traditionally viewed as bystanders in innate immunity, and their role in GA has been largely overlooked. To investigate their potential involvement in GA pathogenesis, we analyzed publicly available single-cell RNA sequencing data (GSE211783) derived from peripheral blood mononuclear cells of three GA patients during both the GA-A and GA-R phases. UMAP-based clustering revealed 12 immune cell subsets (**Figure 3A**), with representative marker gene expression shown in **Supplementary Figures 4A, B**. The distribution of cells between disease phases is shown in **Figure 3B**.

Differential gene expression (DGE) analysis of CD4^+^ naïve T cells identified 28 genes upregulated in GA-A, including pro-inflammatory mediators such as S100A8, S100A9, LYZ, FOS, and FOSB (**Figure 3C**). Conversely, 630 genes, primarily ribosomal protein genes (RPLs, RPSs), were downregulated, indicating suppressed translational capacity. KEGG pathway enrichment analysis based on differentially expressed genes revealed that, compared with GA-R, CD4^+^ naïve T cells from GA-A exhibited upregulation of the IL-17 signaling pathway, whereas pathways such as the FoxO signaling pathway and Insulin signaling pathway were downregulated (**Supplementary Figure 4C**). GSEA found upregulation of Complement, Xenobiotic metabolism, Coagulation, Estrogen response late and Allograft rejection pathways in CD4^+^ naïve T cells from GA-A (**Figure 3D**).

Cell-cell communication analysis revealed that CD4^+^ naïve T cells interacted with multiple immune cell subsets, including CD8^+^ cytotoxic T cells, Granulocyte-like myeloid cells, monocytes, and NK cells (**Figure 3E**). Notably, the differences in intercellular communication patterns between GA-A and GA-R were primarily observed in the interactions between CD4^+^ naïve T cells and Granulocyte-like myeloid cells. Specifically, CD4^+^ naïve T cells from GA-A uniquely exhibited MIF-CXCR2 and ANXA1-FPR1 signaling interactions with Granulocyte-like myeloid cells (**Figure 3F**). These findings suggest that aberrant CD4^+^ naïve T cell-myeloid cell communication, particularly through the MIF-CXCR2 and ANXA1-FPR1 axes, may reflect predicted communication patterns associated with inflammatory and chemotactic programs during acute GA. Notably, CXCR2 and FPR1 were markedly upregulated in Granulocyte-like myeloid cells from GA-A and were identified as characteristic genes of this cell subset (**Supplementary Figure 4D**). In contrast, their corresponding ligands, MIF and ANXA1, although expressed in CD4^+^ naïve T cells, were also detected in other immune cell subsets (**Supplementary Figure 4D**). These findings suggest that CD4^+^ naïve T cells may be involved in specific communication patterns associated with inflammatory programs observed in acute GA.

### Granulocyte-like myeloid cells exhibit enhanced pro-inflammatory and chemotactic signatures during the acute phase of GA

**Figure 4:**
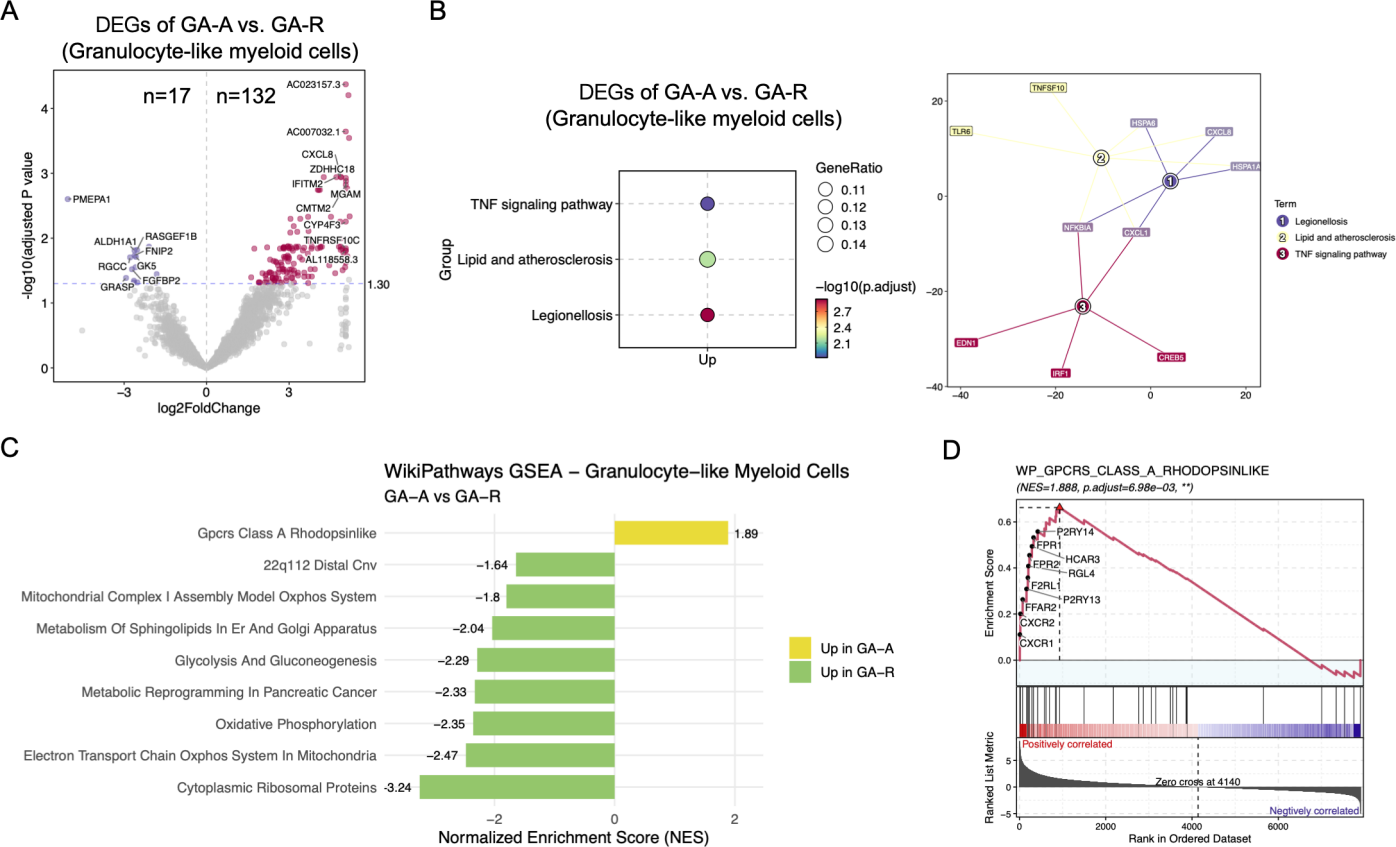
Granulocyte-like myeloid cells exhibit enhanced inflammatory signatures in GA-A. **(A)** Volcano plot showing differentially expressed genes (DEGs) in granulocyte-like myeloid cells between GA-A and GA-R groups. **(B)** KEGG pathway enrichment analysis of upregulated genes in granulocyte-like myeloid cells from GA-A, together with the interaction network of representative core genes from significantly enriched pathways. **(C)** WikiPathways gene set enrichment analysis (GSEA) of granulocyte-like myeloid cells comparing GA-A versus GA-R. Bars indicate normalized enrichment scores (NES) for significantly enriched pathways. Yellow bars represent pathways enriched in GA-A, whereas green bars represent pathways enriched in GA-R. **(D)** GSEA enrichment plot showing enrichment of the “WP_GPCRS_CLASS_A_RHODOPSIN_LIKE” pathway in granulocyte-like myeloid cells from GA-A compared with GA-R. DEGs, differentially expressed genes; GSEA, gene set enrichment analysis; NES, normalized enrichment scores.

Further analysis of Granulocyte-like myeloid cells identified substantial transcriptional alterations between GA-A and GA-R. Differential expression analysis revealed that multiple inflammatory genes, including CXCL8, IFITM2, MGAM, and TNFSF10, were significantly upregulated in GA-A, whereas genes such as ALDH1A1, FGFBP2, and GK5 were downregulated (**Figure 4A**). KEGG enrichment analysis demonstrated that the upregulated genes in GA-A were predominantly enriched in the TNF signaling pathway, lipid and atherosclerosis, and Legionellosis pathways (**Figure 4B**), suggesting enhanced inflammatory activation and innate immune responses in Granulocyte-like myeloid cells during disease activity. Network analysis further showed that genes involved in the TNF signaling pathway, including CXCL1, NFKBIA, IRF1, and CREB5, formed a highly interconnected regulatory module (**Figure 4B**), highlighting the central role of pro-inflammatory signaling in GA-A. Consistently, WikiPathways-based GSEA revealed significant enrichment of the GPCRs Class A Rhodopsin-like signaling pathway in GA-A, whereas pathways related to oxidative phosphorylation, glycolysis/gluconeogenesis, mitochondrial respiratory chain function, and ribosomal activity were relatively enriched in GA-R (**Figure 4C**). More specifically, GSEA indicated marked activation of the GPCRs Class A Rhodopsin-like pathway in GA-A (NES = 1.88, adjusted P = 6.98 × 10^-3), with enrichment of multiple inflammatory chemotactic receptors, including FPR1, CXCR2, CXCR1, FFAR2, and P2RY14 (**Figure 4D**). Taken together, these findings suggest that Granulocyte-like myeloid cells in GA-A exhibit a pronounced pro-inflammatory and chemotactic phenotype accompanied by metabolic reprogramming, which may reflect inflammatory and chemotactic programs associated with immune cell recruitment during active disease.

### Plasma MMP1 and S100A12 levels are elevated in GA-A and correlate with systemic inflammation

**Figure 5:**
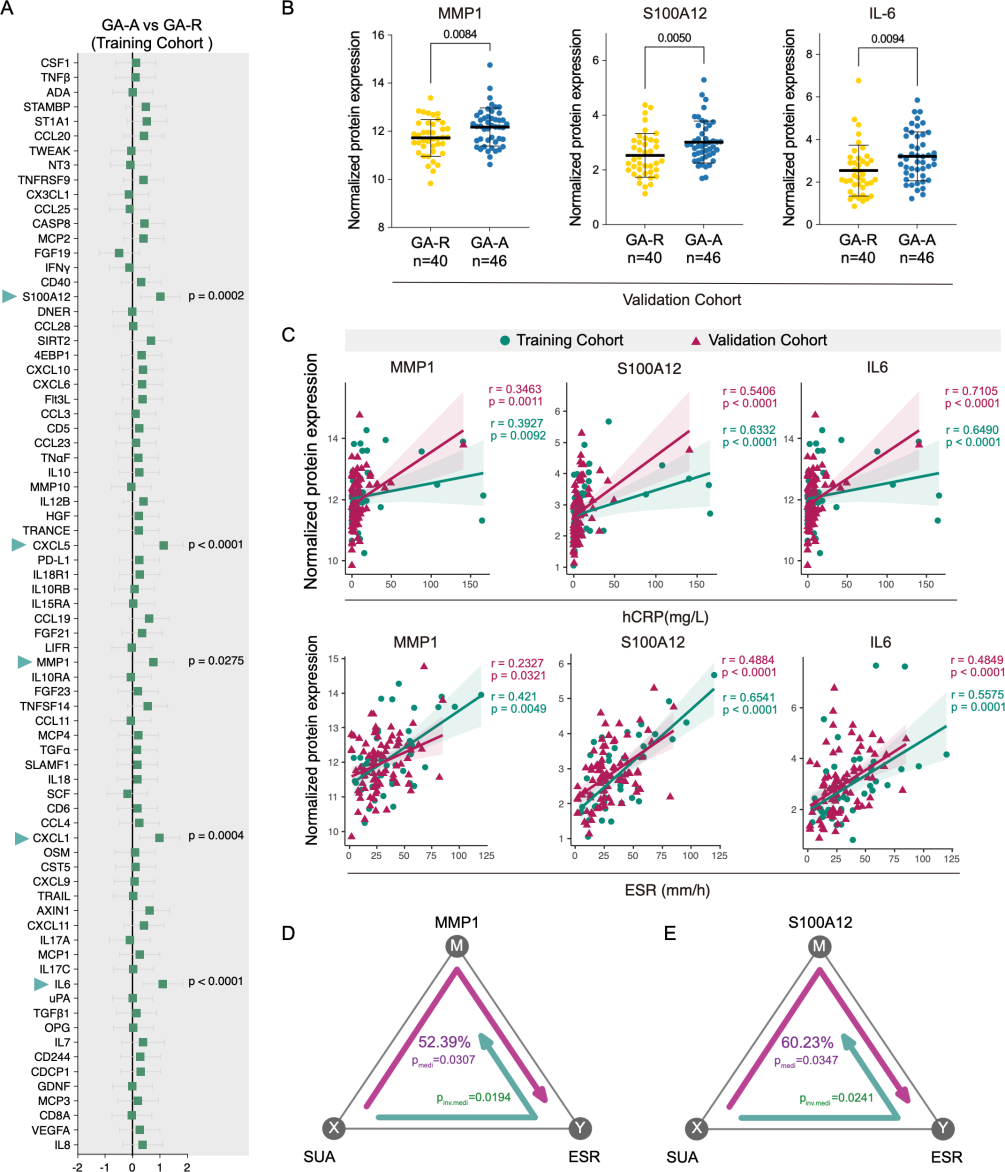
The plasma levels of MMP1 and S100A12 increased in GA-A and positively correlated with hCRP and ESR. **(A)** Forest plot depicting mean differences of NPX between GA-R and GA-A groups in the training cohort. Markers with statistically significant differences are highlighted with adjusted p-values annotated. **(B)** Scatter dot plots showing the NPX levels of MMP1, S100A12, and IL6 in the validation cohort, comparing GA-R (n=40) and GA-A (n=46) groups. Statistical significance is indicated with p-values derived from unpaired t-tests. **(C)** Correlation analyses between NPX levels of immune molecules (MMP1, S100A12, IL6) and clinical inflammatory markers (hCRP and ESR) in both the training (circles) and validation (triangles) cohorts. Spearman correlation coefficients (r) and p-values are shown for each relationship. Regression lines are plotted for visualization, with shaded regions indicating 95% confidence intervals. **(D, E)**. Mediation models evaluating the indirect effect of MMP1 **(D)** and S100A12 **(E)** in the relationship between SUA and ESR. Each triangle diagram depicts the pathways between the predictor (X), mediator (M), and outcome (Y). Percent mediation (indirect effect vs total effect) and corresponding p-values are reported for the mediation model (Path indicated as pink arrow, p_medi_) and invert mediation model (Path indicated as green arrow, p_inv.medi_). NPX, normalized protein expression; hCRP, high-sensitivity C-reactive protein; ESR, erythrocyte sedimentation rate; SUA, serum uric acid; MMP1, matrix metallopeptidase 1; S100A12, S100 calcium binding protein A12.

While prior studies have highlighted associations between serum uric acid (SUA) and inflammatory mediators, the connections between circulating immune proteins and immune cell phenotypes in GA remain largely uncharacterized. To address this gap, we quantified 92 plasma inflammatory proteins using the O-Link platform in both a discovery and an independent validation cohort (GA-A, n = 46; GA-R, n = 40), and explored their relationships with systemic inflammation. In GA-A patients, several pro-inflammatory mediators, including MMP1, S100A12, IL6, FGF21, CCL20, and OSM, were significantly elevated, whereas ST1A1 remained downregulated (**Supplementary Figures 5A, 5C**). Direct comparisons between GA-A and GA-R revealed further increases in MMP1, S100A12, IL6, CXCL1, and CXCL5 during acute inflammation (**Figure 5A**), and these trends were validated in the independent cohort (**Figure 5B**; **Supplementary Table 3**). Spearman correlation analysis demonstrated strong positive associations between plasma MMP1 and S100A12 levels with both hCRP and ESR, reinforcing their role as markers of systemic inflammation (**Figure 5C**). Mediation analysis further revealed that MMP1 and S100A12 mediated 52.39% and 60.23%, respectively, of the association between SUA and ESR elevation (**Figures 5D, E**; **Supplementary Table 7**). These results suggest that MMP1 and S100A12 may represent inflammatory molecules statistically associated with the relationship between hyperuricemia and systemic inflammation in GA.

To further explore whether MSU-induced inflammatory stimulation may be associated with increased MMP1 expression, we performed *in vitro* stimulation experiments using RA-FLS cell line. Notably, stimulation with 200 μg/mL MSU significantly increased MMP1 mRNA expression compared with untreated controls (**Supplementary Figure 5D**). These findings provide additional supportive evidence that MSU-induced inflammatory activation may be associated with MMP1 upregulation. However, further functional studies will be required to determine the precise role of MMP1 in immune cell communication and inflammatory regulation during GA.

### Association between MMP1 and peripheral CD4^+^ naïve T cell abundance in GA

**Figure 6:**
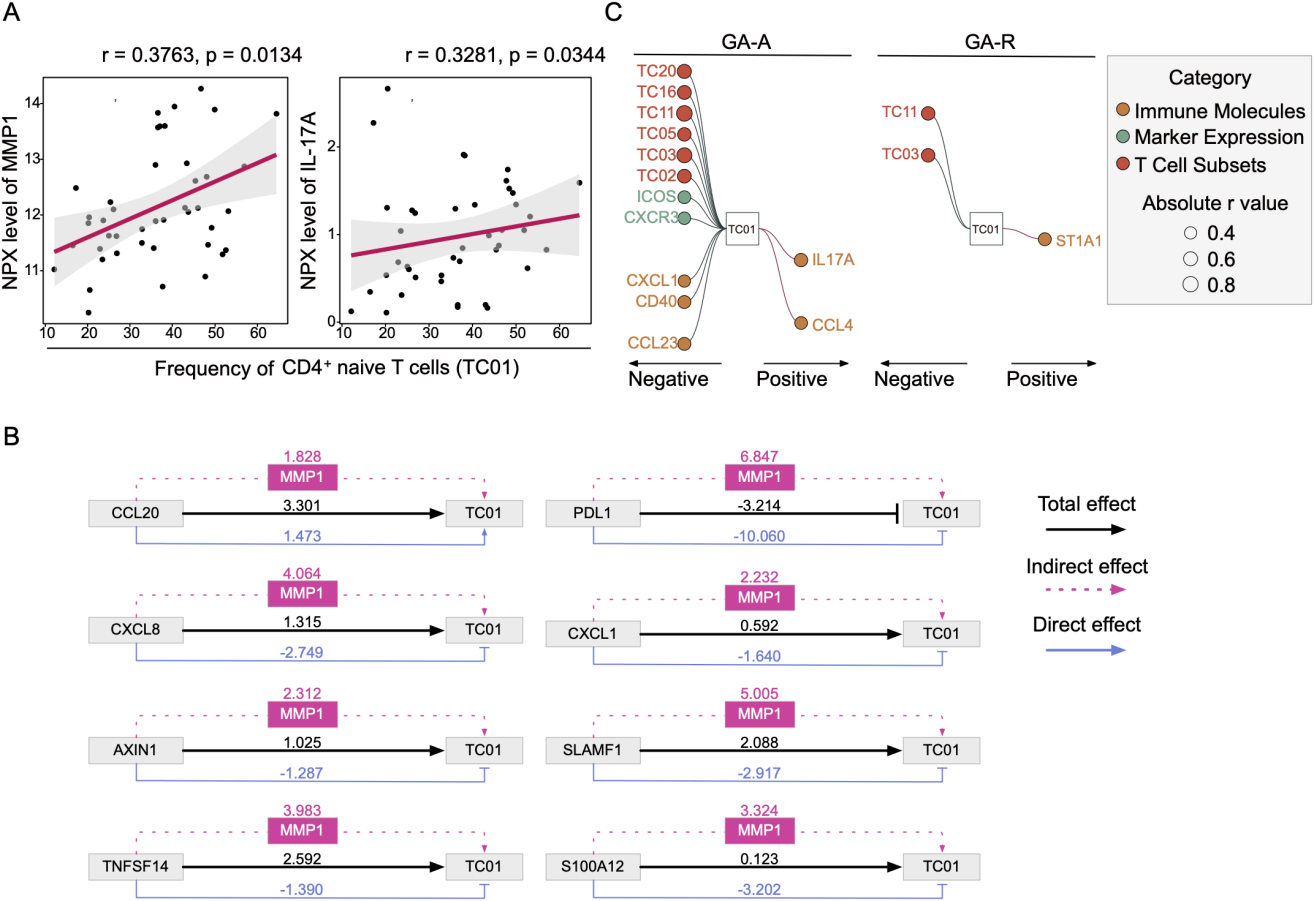
Association between MMP1 and the frequency of CD4^+^ naïve T cells in GA. **(A)** Spearman correlation analysis of frequency of CD4^+^ naïve T cells (TC01) and immune molecules (MMP1 and IL17A). **(B)** Exploratory mediation models evaluating statistical indirect associations involving MMP1 between different immune molecules and the frequency of TC01. **(C)** Spearman correlation analysis of the frequency of TC01 subsets with immune molecules, T cell marker expression, and frequency of other T cell subsets in both GA-A (n=25) and GA-R (n=18) groups. Point size indicates the absolute r value, and color represents the category. PDL1, programmed cell death ligand 1; MMP1, matrix metallopeptidase 1; TNFSF14, TNF superfamily member 14; S100A12, S100 calcium binding protein A12; SLAMF1, signaling lymphocytic activation molecule family member 1; CXCL, C-X-C motif chemokine ligand; ST1A1, sulfotransferase 1A1.

The immunoregulatory effects of soluble inflammatory mediators on peripheral T cell subsets in GA remain poorly defined. To explore this, we examined whether MMP1 and related inflammatory proteins were associated with the abundance of CyTOF-defined CD4^+^ naïve T cells, with particular attention to their potential association patterns. Notably, MMP1 levels were positively correlated with the frequency of CD4^+^ naïve T cells (TC01) (**Figure 6A**), suggesting an association between MMP1-related inflammatory activity and peripheral CD4^+^ naïve T cell remodeling. Exploratory mediation modeling further suggested that MMP1 may statistically mediate associations between several inflammatory factors and TC01 abundance (**Figure 6B**; **Supplementary Table 7**). In these exploratory models, MMP1 was involved in statistical mediation patterns linking CCL20, CXCL8, S100A12, CXCL1, PDL1, and other inflammatory molecules with TC01 abundance (**Figure 6B**).

Additionally, IL17A levels also positively correlated with TC01 cells, with the relationship being more pronounced in GA-A (**Figures 6A, C**). In GA-A patients, TC01 cells exhibited stronger correlation networks with other T cell subsets, in contrast to a relatively uncoupled pattern in GA-R. These findings suggest that CD4^+^ naïve T cells may be involved in systemic immune remodeling during acute GA, potentially in association with Th17-related inflammatory responses.

### Exploratory analysis of MMP1 in 12-week recurrence risk stratification

**Figure 7:**
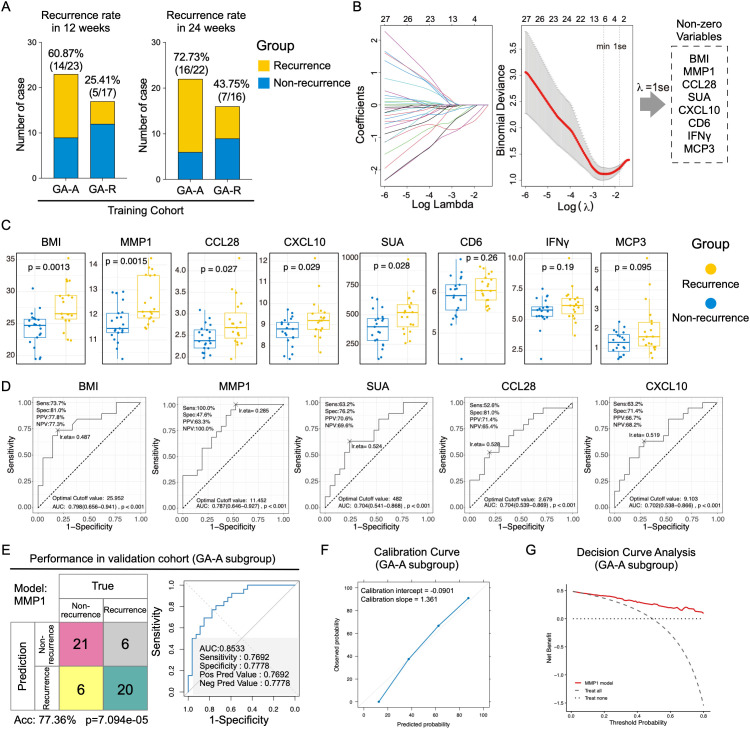
Exploratory analysis of MMP1-associated recurrence risk stratification in GA. **(A)** Stacked bar plots showing the number of patients who experienced recurrence or non-recurrence within 12 and 24 weeks in GA-A and GA-R groups from the training cohort. **(B)** LASSO regression analysis identifying eight non-zero variables associated with recurrence status in the training cohort. **(C)** Boxplots comparing the eight non-zero variables selected by LASSO regression between recurrence and non-recurrence groups. Statistical comparisons were performed using Student’s t-tests. **(D)** ROC curves of five key variables in modeling recurrence and non-recurrence in training cohort, with AUC, 95% confidence intervals, optimal cutoff values, and p-values provided. **(E)** Discriminatory performance of the MMP1-based logistic regression model within the GA-A subgroup in the independent validation cohort. Left panel: confusion matrix. Training cohort, n =23 and validation cohort, n=53. Right panel: ROC curve with corresponding AUC, sensitivity, specificity, PPV, and NPV values. **(F)** Calibration curve analysis of the MMP1-based logistic regression model in the GA-A subgroup validation cohort. The calibration intercept and slope are shown to evaluate agreement between predicted and observed recurrence probabilities. **(G)** Decision curve analysis (DCA) of the MMP1-based recurrence model within the GA-A subgroup. The red line represents the MMP1 model, whereas the dashed lines represent the “treat-all” and “treat-none” strategies. BMI​, body mass index​; SUA​, serum uric acid;​ ROC, ​ receiver operating characteristic​; ​AUC, area under the ROC curve; PPV, positive predictive value; NPV, negative predictive value; DCA, decision curve analysis.

Although models have been developed to distinguish between the acute and remission phases of GA, no reliable tools are currently available to predict short-term relapse. To address this unmet clinical need, we developed and validated a model for predicting 12-week recurrence by integrating proteomic and clinical data. Given that many GA patients experience relapse following an acute episode, we monitored recurrence events at 12- and 24-weeks post-sampling in both the discovery and validation cohorts. GA-A patients exhibited significantly higher recurrence rates at both timepoints compared to GA-R patients (**Figure 7A**; **Supplementary Figure 6A**), highlighting the clinical relevance of identifying early predictors of relapse.

Next, we constructed exploratory models for 12-week recurrence using O-Link-derived plasma protein levels, laboratory indices (SUA, ESR, hCRP), and body mass index (BMI) from the training cohort (GA-A and GA-R pooled, n = 40), followed by validation in an independent cohort (GA-A and GA-R pooled, n = 64). LASSO regression identified eight candidate variables with the strongest association to recurrence status, including BMI, MMP1, CCL28, SUA, CXCL10, CD6, IFN-γ, and MCP3 (**Figure 7B**). Among these, BMI, MMP1, CCL28, SUA, and CXCL10 demonstrated significant differences between recurrence and non-recurrence groups in training cohort (**Figure 7C**). ROC analysis demonstrated that BMI (AUC = 0.798) and MMP1 (AUC = 0.787) showed the strongest individual discriminatory performance (**Figure 7D**). To evaluate model discrimination performance, we built logistic regression models using combinations of the five significant predictors. Notably, the model using MMP1 alone achieved the highest AUC (0.798) and accuracy of 67.19% in the validation cohort (**Supplementary Figures 6B, C**). Validation in an independent cohort further supported the association between elevated MMP1 and recurrence risk (**Supplementary Figure 6D**). To assess model robustness across different machine learning algorithms, we compared logistic regression to Boosted Tree, Decision Tree, K-Nearest Neighbors, and Random Forest models. Logistic regression consistently achieved the best performance, with the highest AUC and accuracy, and the lowest Brier score (**Supplementary Figure 6E**).

Because baseline MMP1 levels differed between GA-A and GA-R patients, we further performed sensitivity analyses restricted to the GA-A subgroup to reduce the potential influence of disease phase on recurrence risk stratification analyses. Notably, within the GA-A subgroup, the MMP1-based model maintained favorable discrimination performance. In the training cohort, the model achieved an AUC of 0.849, while in the independent evaluation cohort, the model showed comparable discrimination ability with an AUC of 0.8533 (**Figure 7E**; **Supplementary Table 8**). Both the AUC and classification accuracy were improved compared with the original pooled GA-A/GA-R model. In addition, calibration analysis was performed in the GA-A subgroup validation cohort to further assess model interpretability. The calibration curve demonstrated acceptable agreement between predicted and observed recurrence probabilities (**Figure 7F**; **Supplementary Table 8**). The calibration intercept was −0.09, indicating minimal systematic overestimation or underestimation of recurrence probabilities, whereas the calibration slope was 1.36, suggesting overall reasonable calibration performance within the current cohort. Moreover, decision curve analysis (DCA) demonstrated that the MMP1-based model provided superior net benefit compared with both the “treat-all” and “treat-none” strategies across a broad range of threshold probabilities (**Figure 7G**), supporting the exploratory relevance of MMP1 for recurrence risk stratification.

Taken together, these findings suggest that elevated MMP1 is associated with increased short-term recurrence risk in GA and may serve as a candidate marker for recurrence stratification. The consistent discrimination performance observed across the current cohorts and multiple modeling approaches suggests that MMP1 may have exploratory relevance for recurrence risk stratification, although further prospective validation in larger multicenter cohorts will be required.

## Discussion

The core pathogenesis of GA has long been attributed to innate immune activation triggered by MSU crystal deposition within joints (1). However, accumulating evidence suggests that adaptive immunity, particularly T cell-mediated responses, may also play a critical role in disease onset and progression (as reviewed in Ref (18)). In this study, our results suggest potential genetic associations between adaptive immune traits and gout risk. In peripheral blood, CD4^+^ naïve T cells showed disease phase-associated remodeling and predicted interactions with granulocyte-like myeloid cells. MMP1 was associated with systemic inflammation, CD4^+^ naïve T cell abundance, and recurrence risk. These findings provide exploratory evidence for immune-associated inflammatory signatures in GA.

We first identified 35 immune cell phenotypes with potential genetic associations with gout, many of which are involved in adaptive immunity. Notably, elevated number of DNT cells, CD127 on CD4^+^ T cells, and HLA-DR on pDCs and DC were associated with increased disease risk, while higher CD45RA expression on CD39^+^ resting Tregs was associated with lower gout risk. Although DNT cells have been relatively understudied in the context of GA, our previous study also observed increased DNT cell abundance in GA (15), consistent with findings from this study. Recent single-cell transcriptomic analyses further suggest that DNT cells may acquire cytotoxic potential via upregulation of TATDN2 and CEACAM21, potentially contributing to joint damage (19). However, these functional hypotheses require validation through molecular and cellular experiments. HLA-DR, a key MHC class II molecule, presents exogenous antigens to CD4^+^ T cells and initiates adaptive immune responses. Its elevated expression on DCs correlating with GA progression underscores the involvement of adaptive immunity.

CD39 functions as an ectonucleotidase that initiates the conversion of extracellular nucleotides, such as ATP and ADP, into AMP (20, 21). This enzymatic activity represents a critical step in the generation of extracellular adenosine, a potent immunosuppressive mediator. Its role is particularly significant in inflammatory conditions, where excessive ATP release from damaged tissues can drive immune activation. CD39^+^ Tregs can counteract this process, thereby promoting inflammation resolution and preventing tissue damage (21). These findings challenge the traditional paradigm that places innate immunity at the center of GA pathophysiology and suggest that adaptive immune dysregulation, particularly impaired immune tolerance, may also be involved.

We observed marked shifts in peripheral T cell composition in GA, with four subsets elevated and three, including Tregs, reduced compared to HC. This remodeling likely reflects an immune adaptation to the inflammatory state in GA. The decline in Tregs, together with the MR findings, may reflect altered immunoregulatory states associated with GA.

We next compared T cell profiles between GA-A and GA-R. Although few subsets differed, CD4^+^ naïve T cells stood out as both the most abundant T cell population and the only subset significantly increased during the acute phase. CD4^+^ naïve T cells can differentiate into various Th subtypes depending on the cytokine milieu, thus influencing the Th1/Th2 and Th17/Treg balance. In GA, elevated levels of Th17- promoting cytokines such as IL-6 and IL-23 have been reported (22, 23), supporting a pro-inflammatory differentiation bias. This aligns with findings of Th17 enrichment in inflamed joints (10). Therefore, the rise in CD4^+^ naïve T cells during flares may be associated with altered peripheral T-cell differentiation states during acute inflammation. Whether CD4^+^ naïve T cells themselves contribute directly to inflammation remains unclear. We revealed that in GA-A, these cells upregulated genes involved in complement and inflammatory pathways, including S100A8 and S100A9. These alarmins activate APCs via TLR4 or RAGE, triggering NF-κB and MAPK pathways and promoting secretion of IL-1β and IL-6, thus indirectly enhancing Th1/Th17 differentiation (24).

Unexpectedly, we also identified enhanced communication between CD4^+^ naïve T cells and granulocyte-like myeloid cells via the predicted MIF-CXCR2 and ANXA1-FPR1 signaling interactions in GA-A. CXCR2 and FPR1 were markedly enriched in granulocyte-like myeloid cells, whereas MIF and ANXA1 were expressed across multiple immune cell subsets rather than being uniquely derived from CD4^+^ naïve T cells, suggesting that these inflammatory communication patterns may primarily reflect activation programs within the granulocyte-like myeloid compartment. Importantly, although these cells could not be definitively classified as canonical neutrophils due to the limitations of PBMC-based single-cell sequencing, they exhibited multiple granulocytic and neutrophil-associated features, including expression of FCGR3B, CXCR2, CSF3R, CEACAM8, S100A8, and S100A9. Previous studies have established that neutrophils are major effector cells during acute gout flares and contribute to inflammatory amplification through chemokine-mediated recruitment, NETs formation, inflammasome activation, and IL-1β release (25–28). Notably, experimental studies in mouse models of acute gout have also implicated the MIF–CXCR2 signaling axis in neutrophil recruitment and inflammatory amplification, supporting the biological relevance of the predicted MIF-related communication patterns identified in our analysis (29). Consistent with these neutrophil-associated inflammatory programs, CXCL5 and CXCL12 were elevated in GA-A plasma in our study, while IL1B-related inflammatory pathways were enriched within the granulocyte-like myeloid cell population (30, 31). In addition, S100A9, which was upregulated in CD4^+^ naïve T cells, has previously been implicated in enhancing CXCR2-dependent granulocytic chemotaxis and inflammatory recruitment (32). Together, these observations raise the possibility that peripheral CD4^+^ naïve T cell remodeling may be associated with inflammatory communication networks involving granulocyte-like myeloid cells during acute GA.

Proteomic profiling identified MMP1, S100A12, and IL6 as significantly elevated in GA-A, each correlating positively with hCRP and ESR. MMP1 and S100A12 were also identified as statistical mediators linking SUA to ESR in exploratory mediation models, suggesting that they may reflect inflammatory pathways associated with hyperuricemia. Among them, MMP1 was particularly notable: its levels correlated with CD4^+^ naïve T cell proportions and showed statistical mediation patterns with other immune mediators. Although limited research has addressed the mechanistic role of MMP1 in GA, previous studies have reported its elevation in patient serum and synovial fluid and proposed its exploratory inclusion in diagnostic models alongside VEGFA and OSM (32). MMP1 showed exploratory potential for short-term recurrence risk stratification, although its potential relevance for recurrence risk stratification requires prospective validation. Nonetheless, the pathways linking MMP1 with CD4^+^ naïve T cell remodeling and disease pathology remain to be elucidated through further molecular studies.

In this integrative multi-omics study, we identified peripheral CD4^+^ naïve T cell expansion and MMP1-associated inflammatory signatures as potential features of acute GA. Our findings suggest that CD4^+^ naïve T cell remodeling in peripheral blood may reflect systemic immune alterations during acute flares and may be associated with inflammatory programs involving granulocyte-like myeloid cells. In addition, MMP1 was significantly associated with systemic inflammatory markers, CD4^+^ naïve T cell abundance, and short-term recurrence risk, highlighting its exploratory relevance as a candidate marker in GA.

There are several limitations to this study. First, the associations between MMP1 and CD4^+^ naïve T cell abundance were derived from cross-sectional observational data and therefore cannot establish biological relationships or temporal ordering. Although mediation analysis suggested potential statistical indirect associations, alternative explanations, including shared upstream inflammatory drivers, cannot be excluded. In addition, no functional perturbation experiments or coculture systems were performed to determine whether MMP1 directly regulates CD4^+^ naïve T cell biology or inflammatory communication in GA. Second, the immune profiling analyses were performed using peripheral blood samples rather than synovial fluid or joint tissue. Therefore, the observed CD4^+^ naïve T cell remodeling and predicted cell-cell communication patterns may reflect systemic immune alterations rather than local joint-specific inflammatory mechanisms during acute gout flares. Third, the granulocyte-like myeloid cell populations identified in the PBMC-derived scRNA-seq dataset could not be definitively classified as canonical neutrophils because conventional high-density neutrophils are typically underrepresented during PBMC isolation. Although re-annotation and marker-based curation were performed, the precise identity of these cells remains to be further validated using dedicated granulocyte-preserving protocols. Fourth, the different analytical layers used in this study were derived from populations with different ancestry backgrounds. Specifically, the Mendelian randomization analyses were based predominantly on European ancestry GWAS datasets, whereas the CyTOF, proteomic, and clinical cohorts consisted of Chinese participants. Therefore, these findings should not be interpreted as forming a direct causal-to-clinical validation chain, and ancestry-specific differences in genetic architecture and immune traits may influence the generalizability of the results. Finally, the exploratory recurrence risk analyses were conducted in relatively small cohorts, and structural damage-related parameters such as imaging-based erosion scores or tophus burden were not systematically available. Therefore, the potential clinical relevance and biological relevance of MMP1 for long-term disease progression and joint damage require further validation in larger prospective multicenter studies.

## Conclusion

In summary, we identified consistent immune-associated patterns related to acute GA and recurrence risk. Peripheral CD4^+^ naïve T cells and MMP1 emerged as potentially relevant components of the inflammatory landscape observed during acute flares and recurrence. While the mechanistic relationships among these immune features remain incompletely understood and require further validation using synovial tissue, ancestry-matched cohorts, and functional experimental studies, our findings provide a framework for future investigations into immune-associated candidate markers and inflammatory pathways in GA.

## Data Availability

The datasets presented in this study can be found in online repositories. The names of the repository/repositories and accession number(s) can be found in the article/**Supplementary Material**.

## Supplementary material

Supplementary Figure 1 Reversed Mendelian randomization analysis result. **(A)** Reversed MR analysis assessing genetic associations of gout with 35 immune cell phenotypes using the inverse-variance weighted (IVW) method.

Supplementary Figure 2 CyTOF gating strategy for immune cell analysis. A. Raw CyTOF FCS files were first normalized and debarcoded prior to downstream analysis. Sequential manual gating was performed in FlowJo. Cellular events were initially identified based on high Ir191 DNA and Ir193 DNA signals to exclude debris and non-cellular impurities. Viable cells were subsequently selected by excluding cisplatin-positive events using the Pt194Di (194 cisplatin) channel. Doublets and cell aggregates were removed using Event Length gating to retain singlet cells. Residual normalization beads were excluded using the Ce140Di channel. The resulting viable singlet immune cells were used for subsequent downstream analyses.

Supplementary Figure 3 The immune landscape of GA at different stages. **(A)** tSNE plot visualizing key cell markers expression in immune cells (CD66b^-^CD45^+^) with color-coded clusters representing distinct cell subpopulations. **(B)** Boxplots demonstrating the relative frequencies of major immune cell subpopulation across GA-A (n=25), GA-R (n=22), and HC (n=9). Statistical significance was evaluated using the Kruskal-Wallis test, with corresponding p-values indicated for each comparison. **(C)** Volcano plots illustrating the differential expression of markers of major immune cell subpopulations in GA-A and GA-R groups. The Y-axis represents the p-values derived from T-test analyses, while the X-axis shows the intensity differences of each marker (calculated as GA-A minus GA-R). Markers with increased expression in GA-A are highlighted in pink, whereas those with decreased expression are depicted in light blue. GA-A, gout patients at acute stage; GA-R, gout patients at remission stage; HC, healthy individuals; TC, T cell cluster; TCM, central memory T cells; TEM, effector memory T cells; NKT, natural killer T cells; DNT, CD4 and CD8 double negative T cells; DPT, CD4 and CD8 double positive T cells.

Supplementary Figure 4 Additional analyses and marker characterization of single-cell transcriptomic data. **(A)** Dot plot showing representative marker genes used for manual annotation of immune cell subsets in the scRNA-seq dataset. Dot size indicates the percentage of cells expressing each marker, and color intensity indicates average expression level. **(B)** Dot plot showing the expression patterns of selected immune and granulocyte-associated marker genes across annotated immune cell subsets. Granulocyte-like inflammatory myeloid cells exhibited expression of FCGR3B, CSF3R, CXCR2, CEACAM8, S100A8, and S100A9, consistent with granulocyte-associated inflammatory features. **(C)** KEGG pathway enrichment analysis of differentially expressed genes in scRNA-seq-defined CD4^+^ naïve T cells comparing GA-A versus GA-R. Dot size represents gene ratio, and color intensity indicates adjusted p values. **(D)** UMAP feature plots showing the expression and spatial distribution of CXCR2, ANXA1, FPR1, and MIF in GA-A and GA-R samples. Red ellipses highlight granulocyte-like myeloid cell- and CD4^+^ naïve T cell-enriched regions.

Supplementary Figure 5 Differential immune signatures of GA-A, GA-R, and HC. **(A)** A heatmap displaying scaled NPX levels of immune molecules measured in plasma samples from GA-A, GA-R, and HC participants. Purple indicates higher expression, while green represents lower expression. **(B)** Forest plot illustrating the mean differences in NPX levels between HC and GA-R groups in the training cohort. Statistically significant markers are annotated with adjusted p-values. **(C)** Forest plot illustrating the mean differences in NPX levels between HC and GA-A groups in the training cohort. Statistically significant markers are annotated with adjusted p-values. **(D)** Relative MMP1 mRNA expression in RA-FLS stimulated with different concentrations of monosodium urate (MSU) crystals for 24 hours. Data are presented as mean ± SEM. Statistical significance was determined using one-way ANOVA with *post hoc* multiple comparison analysis.

Supplementary Figure 6 Exploratory performances of GA recurrence risk stratification models. **(A)** Stacked bar plots depict the number of patients experienced recurrence (yellow) and non-recurrence (blue) in12 weeks and 24 weeks in GA-A and GA-R groups from validation cohort. **(B)** Line plot illustrating changes in model performance (AUC in blue, Accuracy in yellow) across different combinations of predictors in logistic regression-based recurrence risk stratification models, evaluated in the validation cohort. **(C)** Discriminatory performance of the MMP1-based logistic regression model in the pooled GA-A/GA-R validation cohort. Left panel: confusion matrix. Right panel: ROC curve with corresponding AUC value. (D) Expression levels of MMP1 in plasma samples from recurrence and non-recurrence groups within the validation cohort. Mann-Whitney U test was used for statistical comparison. **(E)** Comparison of five machine learning models (Boost Tree, Decision Tree, Logistic Regression, Nearest Neighbor, and Random Forest) using MMP1 as the sole variable for exploratory GA recurrence risk stratification. Bar plots show model performance in terms of accuracy, Brier class score, and AUC. Error bars represent standard deviations across resampling iterations. **(F)** ROC curve analysis of MMP1 for recurrence risk stratification in the pooled GA-A/GA-R cohort. AUC, optimal cutoff value, sensitivity, specificity, positive predictive value (PPV), and negative predictive value (NPV) are shown.
