# Supplementary material for: Peripheral CD4+ naïve T cell remodeling and MMP1-associated inflammatory signatures in acute gouty arthritis
Source: Front Immunol. 2026 May 26;17:1828679. doi: 10.3389/fimmu.2026.1828679 (PMC13246714; doi:10.3389/fimmu.2026.1828679)
Supplement: Supplementary Figure 1 — Reversed Mendelian randomization analysis result. (A) Reversed MR analysis assessing genetic associations of gout with 35 immune cell phenotypes using the inverse-variance weighted (IVW) method. [file Supplementaryfile1.pdf]

Supplementary figures

A

Reversed MR analysis （Gout==>Immunocyte phenotypes)

Immunocyte phenotypes	Method	NSNP	p value	OR(95%CI)
CD45RA on CD39+ resting Treg	Inverse variance weighted	11	0.0033	0.92 (0.87−0.97)
HLA DR on plasmacytoid DC	Inverse variance weighted	33	0.1313	0.97 (0.94−1.01)
CD19 on IgD− CD38−	Inverse variance weighted	33	0.1073	0.97 (0.94−1.01)
HLA DR on DC	Inverse variance weighted	33	0.2289	0.98 (0.94−1.01)
CD27 on CD24+ CD27+	Inverse variance weighted	33	0.2685	0.98 (0.95−1.01)
CD28 on CD39+ CD4+	Inverse variance weighted	33	0.3736	0.98 (0.94−1.02)
CD14+ CD16− monocyte %monocyte	Inverse variance weighted	33	0.2847	0.98 (0.95−1.01)
FSC−A on NK	Inverse variance weighted	33	0.3779	0.98 (0.95−1.02)
CD20 on B cell	Inverse variance weighted	33	0.4588	0.99 (0.95−1.03)
Naive CD4+ %T cell	Inverse variance weighted	33	0.5072	0.99 (0.95−1.03)
CD11c+ HLA DR++ monocyte AC	Inverse variance weighted	33	0.5138	0.99 (0.95−1.03)
CD127 on CD4+	Inverse variance weighted	11	0.6760	0.99 (0.94−1.04)
CD62L− myeloid DC AC	Inverse variance weighted	33	0.7660	0.99 (0.96−1.03)
CD25 on unsw mem	Inverse variance weighted	33	0.7843	1.00 (0.96−1.03)
CD86 on myeloid DC	Inverse variance weighted	33	0.7926	1.00 (0.96−1.03)
CD25 on IgD+ CD24+	Inverse variance weighted	33	0.8565	1.00 (0.96−1.04)
SSC−A on CD4+	Inverse variance weighted	33	0.8510	1.00 (0.96−1.03)
CD39 on CD39+ secreting Treg	Inverse variance weighted	33	0.9207	1.00 (0.96−1.03)
CD11c on granulocyte	Inverse variance weighted	33	0.9732	1.00 (0.96−1.04)
CCR2 on CD14− CD16+ monocyte	Inverse variance weighted	33	0.8831	1.00 (0.97−1.04)
CD16−CD56 on HLA DR+ NK	Inverse variance weighted	33	0.8735	1.00 (0.97−1.04)
DP (CD4+CD8+) AC	Inverse variance weighted	33	0.7601	1.01 (0.97−1.04)
CD20 on IgD+ CD38− naive	Inverse variance weighted	33	0.8443	1.01 (0.95−1.06)
CD8 on naive CD8br	Inverse variance weighted	33	0.7274	1.01 (0.97−1.04)
CD45RA+ CD28− CD8br %T cell	Inverse variance weighted	33	0.9423	1.01 (0.83−1.23)
TD DN (CD4−CD8−) %DN	Inverse variance weighted	33	0.6577	1.01 (0.97−1.04)
CD16 on CD14− CD16+ monocyte	Inverse variance weighted	33	0.6309	1.01 (0.98−1.04)
IgD+ CD24+ %B cell	Inverse variance weighted	33	0.5934	1.01 (0.98−1.04)
CD62L on granulocyte	Inverse variance weighted	33	0.5975	1.01 (0.98−1.04)
CD40 on CD14+ CD16− monocyte	Inverse variance weighted	33	0.4436	1.01 (0.98−1.05)
IgD− CD38dim %lymphocyte	Inverse variance weighted	33	0.3208	1.02 (0.98−1.05)
CD25 on IgD+ CD38−	Inverse variance weighted	33	0.3671	1.02 (0.98−1.05)
DN (CD4−CD8−) AC	Inverse variance weighted	33	0.2939	1.02 (0.99−1.05)
CD16+ monocyte %monocyte	Inverse variance weighted	33	0.2753	1.02 (0.99−1.05)
IgD− CD24− %B cell	Inverse variance weighted	33	0.1775	1.02 (0.99−1.06)
CD11b on Gr MDSC	Inverse variance weighted	12	0.4905	1.03 (0.95−1.11)

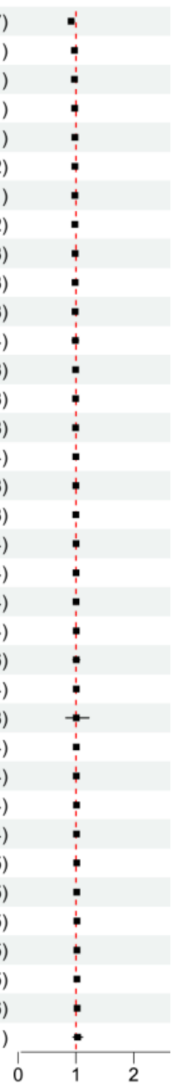

Figure S1. Reversed Mendelian randomization analysis result

A. Reversed MR analysis assessing genetic associations of gout with 35 immune cell phenotypes using the inverse-variance weighted (IVW) method.

A

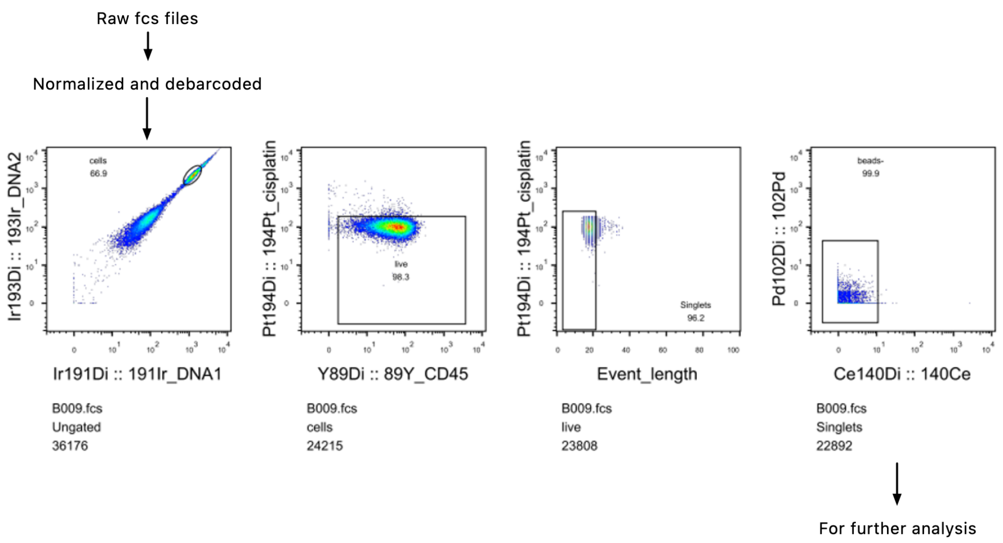

Figure S2. CyTOF gating strategy for immune cell analysis.

A. Raw CyTOF FCS files were first normalized and debarcoded prior to downstream analysis. Sequential manual gating was performed in FlowJo. Cellular events were initially identified based on high Ir191 DNA and Ir193 DNA signals to exclude debris and non-cellular impurities. Viable cells were subsequently selected by excluding cisplatin-positive events using the Pt194Di (194 cisplatin) channel. Doublets and cell aggregates were removed using Event Length gating to retain singlet cells. Residual normalization beads were excluded using the Ce140Di channel. The resulting viable singlet immune cells were used for subsequent downstream analyses.

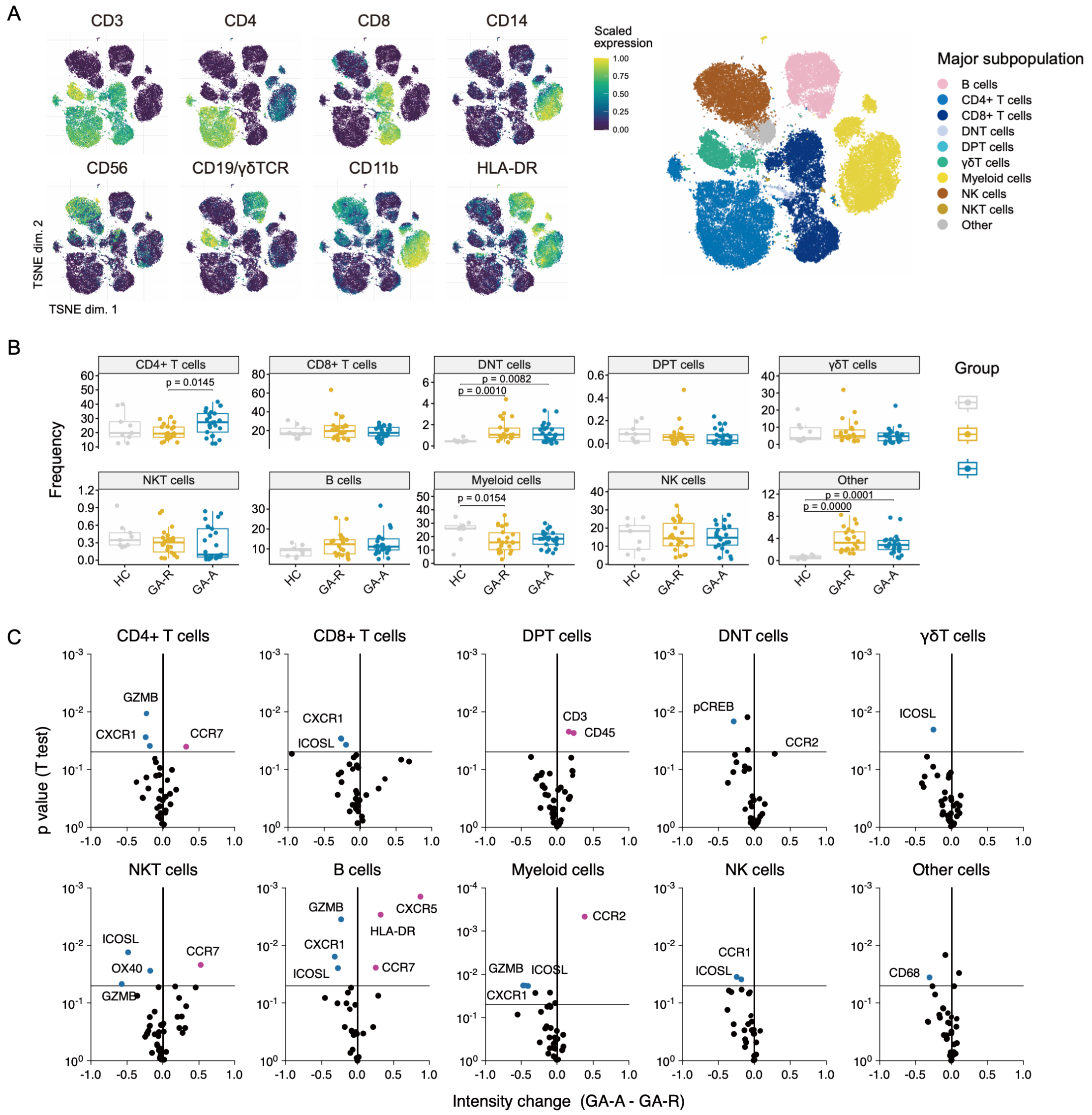

Figure S3. The immune landscape of GA at different stages

A. tSNE plot visualizing key cell markers expression in immune cells ($CD66b^{-}CD45^{+}$) with color-coded clusters representing distinct cell subpopulations.

B. Boxplots demonstrating the relative frequencies of major immune cell subpopulation across GA-A (n=25), GA-R (n=22), and HC (n=9). Statistical significance was evaluated using the Kruskal-Wallis test, with corresponding p-values indicated for each comparison.

C. Volcano plots illustrating the differential expression of markers of major immune cell subpopulations in GA-A and GA-R groups. The Y-axis represents the p-values derived from T-test analyses, while the X-axis shows the intensity differences of each marker (calculated as GA-A minus GA-R). Markers with increased expression in GA-

A are highlighted in pink, whereas those with decreased expression are depicted in light blue.

Abbreviations: GA-A, gout patients at acute stage; GA-R, gout patients at remission stage; HC, healthy individuals; TC, T cell cluster; TCM, central memory T cells; TEM, effector memory T cells; NKT, natural killer T cells; DNT, CD4 and CD8 double negative T cells; DPT, CD4 and CD8 double positive T cells.

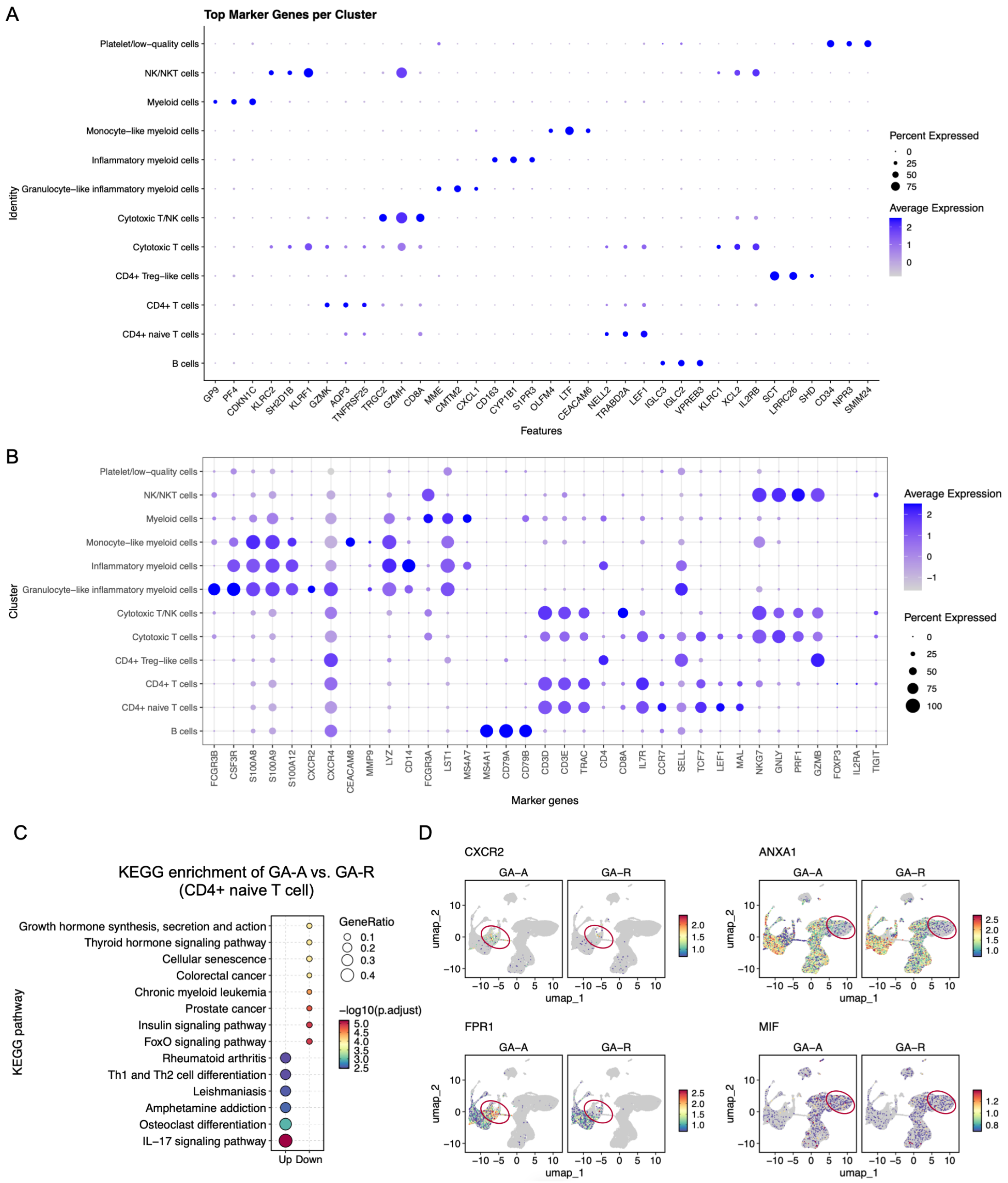

Figure S4. Additional analyses and marker characterization of single-cell transcriptomic data.

A. Dot plot showing representative marker genes used for manual annotation of immune cell subsets in the scRNA-seq dataset. Dot size indicates the percentage of cells expressing each marker, and color intensity indicates average expression level.

B. Dot plot showing the expression patterns of selected immune and granulocyte-associated marker genes across annotated immune cell subsets. Granulocyte-like inflammatory myeloid cells exhibited expression of FCGR3B, CSF3R, CXCR2,

CEACAM8, S100A8, and S100A9, consistent with granulocyte-associated inflammatory features.

C. KEGG pathway enrichment analysis of differentially expressed genes in scRNA-seq-defined $CD4^+$ naïve T cells comparing GA-A versus GA-R. Dot size represents gene ratio, and color intensity indicates adjusted p values.

D. UMAP feature plots showing the expression and spatial distribution of CXCR2, ANXA1, FPR1, and MIF in GA-A and GA-R samples. Red ellipses highlight granulocyte-like myeloid cell- and $CD4^+$ naïve T cell-enriched regions.

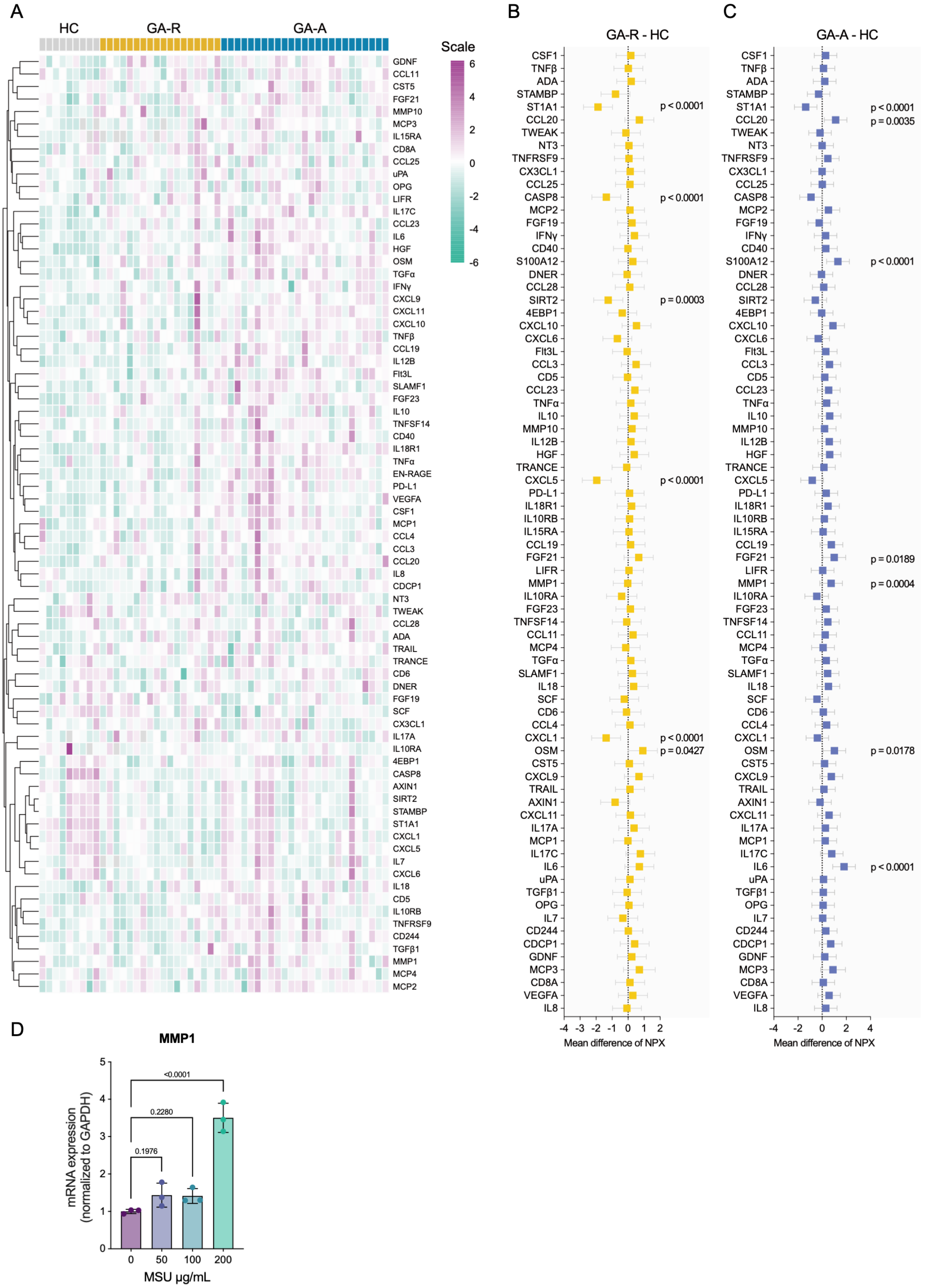

Figure S5. Differential immune signatures of GA-A, GA-R, and HC.

A. A heatmap displaying scaled NPX levels of immune molecules measured in plasma samples from GA-A, GA-R, and HC participants. Purple indicates higher expression, while green represents lower expression.

B. Forest plot illustrating the mean differences in NPX levels between HC and GA-R groups in the training cohort. Statistically significant markers are annotated with adjusted p-values.

C. Forest plot illustrating the mean differences in NPX levels between HC and GA-A groups in the training cohort. Statistically significant markers are annotated with adjusted p-values.

D. Relative MMP1 mRNA expression in RA-FLS stimulated with different concentrations of monosodium urate (MSU) crystals for 24 hours. Data are presented as mean ± SEM. Statistical significance was determined using one-way ANOVA with post hoc multiple comparison analysis.

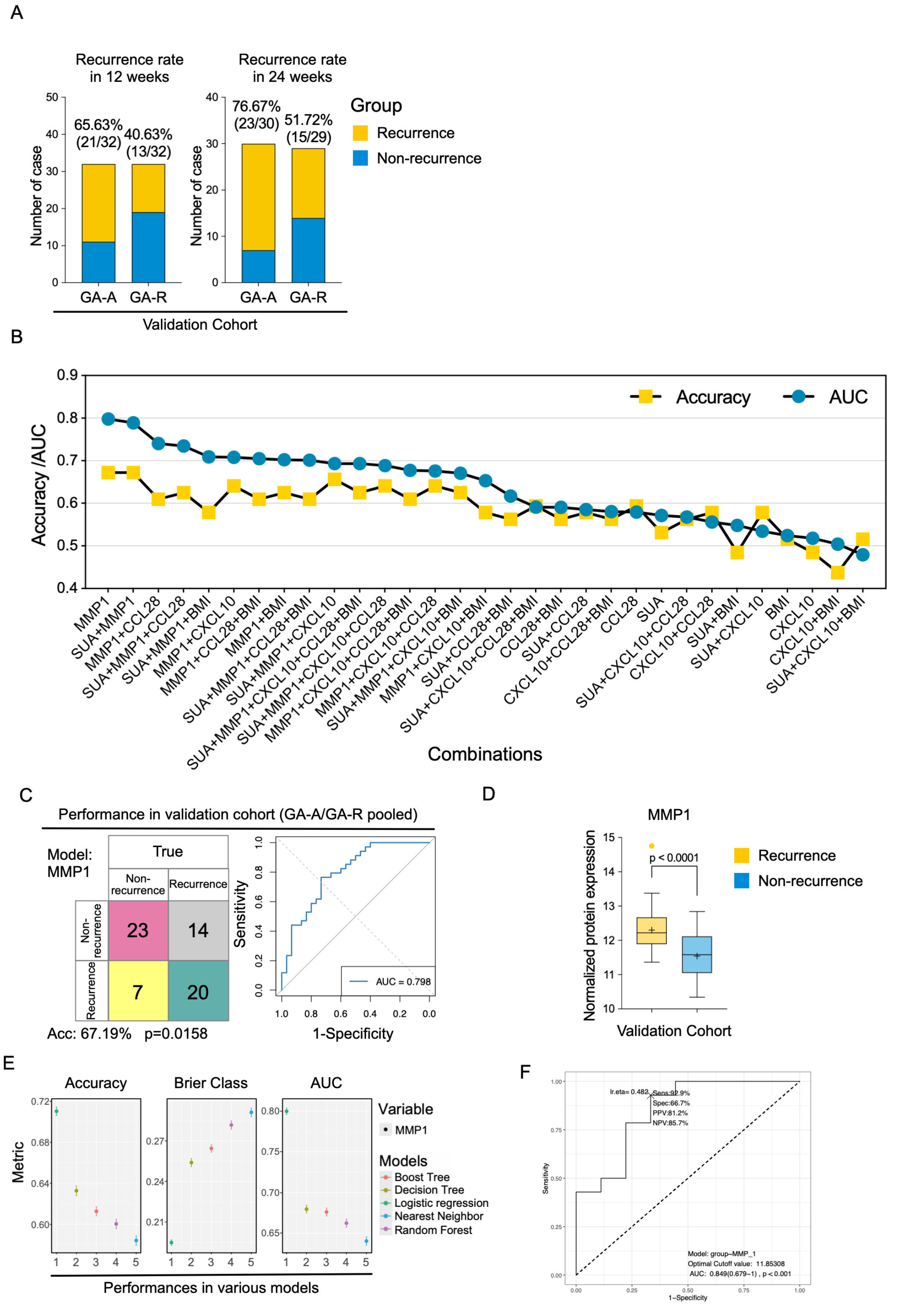

Figure S6. Exploratory performances of GA recurrence risk stratification models.

A. Stacked bar plots depict the number of patients experienced recurrence (yellow) and non-recurrence (blue) in12 weeks and 24 weeks in GA-A and GA-R groups from validation cohort.

B. Line plot illustrating changes in model performance (AUC in blue, Accuracy in yellow) across different combinations of predictors in logistic regression-based recurrence risk stratification models, evaluated in the validation cohort.

C. Discriminatory performance of the MMP1-based logistic regression model in the pooled GA-A/GA-R validation cohort. Left panel: confusion matrix. Right panel: ROC curve with corresponding AUC value.

D. Expression levels of MMP1 in plasma samples from recurrence and non-recurrence groups within the validation cohort. Mann-Whitney U test was used for statistical comparison.

E. Comparison of five machine learning models (Boost Tree, Decision Tree, Logistic Regression, Nearest Neighbor, and Random Forest) using MMP1 as the sole variable for exploratory GA recurrence risk stratification. Bar plots show model performance in terms of accuracy, Brier class score, and AUC. Error bars represent standard deviations across resampling iterations.

F. ROC curve analysis of MMP1 for recurrence risk stratification in the pooled GA-A/GA-R cohort. AUC, optimal cutoff value, sensitivity, specificity, positive predictive value (PPV), and negative predictive value (NPV) are shown.
